# Direct observation of Notch signaling–induced transcription hubs mediating gene-expression responses

**DOI:** 10.1126/sciadv.aea5664

**Published:** 2026-03-06

**Authors:** Carmen Santa-Cruz Mateos, Charalambos Roussos, F. Javier de Haro Arbona, Julia Falo-Sanjuan, Sarah Bray

**Affiliations:** Department of Physiology Development and Neuroscience, University of Cambridge, Downing Street, Cambridge CB2 3DY, UK.

## Abstract

Developmental decisions rely on cells making accurate transcriptional responses to signals they receive, as with Notch pathway activity. Local condensates or transcription factor hubs are a proposed mechanism for facilitating gene activation by nuclear complexes. To investigate their importance in endogenous Notch signaling, we deployed multicolor live imaging to measure Notch transcription-complex enrichment at a target gene locus in combination with the transcription dynamics. The coactivator Mastermind (Mam) was present in signaling-dependent nuclear foci during Notch active developmental stages. Tracking these highly dynamic Mam hubs together with transcription in the same nucleus revealed that their appearance precedes and correlates with the profile of transcription and becomes stabilized if transcription is inhibited. Manipulations to signaling levels had concordant effects on hub intensities and transcription profiles, altering their probability and amplitude. Together, the results argue that signaling induces the formation of transcription hubs whose properties are instrumental in the quantitative gene-expression response to Notch activation.

## Teaser

Multicolor live imaging reveals Notch transcription-complex enrichment at a gene locus, which precedes and informs transcription.

## INTRODUCTION

To make and organize different tissues, cells decipher information from developmental signaling pathways. Transmitting this information accurately, so that cell-surface signals are translated into correct transcriptional responses, is of critical importance. For example, dosage and dynamics of Notch activity are fundamentally important for developmental decisions for sustaining stem cells and for tissue homeostasis (*1*–*5*). Misregulation underlies many diseases including cancers (*6*–*8*). Signaling is normally initiated when transmembrane ligands on adjacent cells interact with the Notch receptor at the membrane. This brings about proteolytic cleavages to release the Notch intracellular domain (NICD), which forms a tripartite complex with the DNA binding protein CSL and the coactivator Mastermind (Mam) (*9*–*11*). The tripartite complex is recruited directly to regulated gene loci, where it promotes their transcription (*12*–*14*). As there is no amplification step, the numbers of active transcription complexes directly relate to the number of receptors activated and it remains to be established how they efficiently and quantitatively elicit target gene transcription.

One model is that Notch transcription complexes form hubs, local zones of high-density clustering that facilitate the recruitment of transcription machinery and RNA polymerase II (Pol II) to direct transcription (*15*, *16*). Hubs or condensates have been detected in a range of contexts where their formation relies on protein-DNA and multivalent protein-protein interactions, often involving low-complexity regions (*17*–*20*). Broadly, they can be described as nonstoichiometric assemblies, which drive biological functions by transiently increasing local concentrations (*21*, *22*). Although initially detected under conditions of protein overexpression, transcription factor hubs are increasingly observed under physiological conditions, making it plausible that they perform an important step in transcriptional regulation (*23*–*26*). Recent studies suggest that hub densities correlate with the transcriptional output from gene loci (*27*).

There is mounting evidence that condensation of nuclear hormone receptors and co-regulators plays an important role in transcriptional regulation (*28*, *29*). Assembly of nuclear condensates containing yes-associated protein (YAP) and transcription coactivators has also been seen in cells, where it is modulated by Hippo signaling or mechanical changes (*30*). Similarly, using live imaging approaches, we previously observed a hub-like enrichment of Notch transcription complexes around a target locus in a tissue where ectopic Notch activity was induced (*15*, *16*). As with the YAP condensates, the zone of enrichment concentrated additional factors that included Mediator and Pol II in those nuclei where transcription was occurring (*15*, *30*). It is thus plausible that such hubs play an integral part in signaling responses.

To explore the role of transcription hubs in Notch signaling, we focus here on a biological context where the Notch pathway is endogenously active, the *Drosophila* follicular epithelium. Through in vivo live imaging of endogenous members of Notch transcription complexes together with a method for labeling the Notch-regulated *Enhancer of split-*Complex [*E(spl)*-*C*], we could detect localized transcription hubs in the stages when Notch signaling is occurring. To investigate the role of these hubs in mediating the response to Notch, we tracked the co-activator Mam, a component of the Notch active transcription complexes, at the same time as measuring responding gene-expression profiles using the MS2/MCP system with two Notch targets. This revealed that the Mam transcription hubs are dynamic and reach their maximal intensity just before transcription initiation. The hub intensity is altered by changes in Notch activity, indicating that they are signaling induced, and the consequential effects on transcription argue that gene-specific Mam transcription hubs have a key role in coordinating the probability and levels of gene activity in response to Notch activation.

## RESULTS

### Mam hubs are present during the Notch active time window

**Fig. 1.**
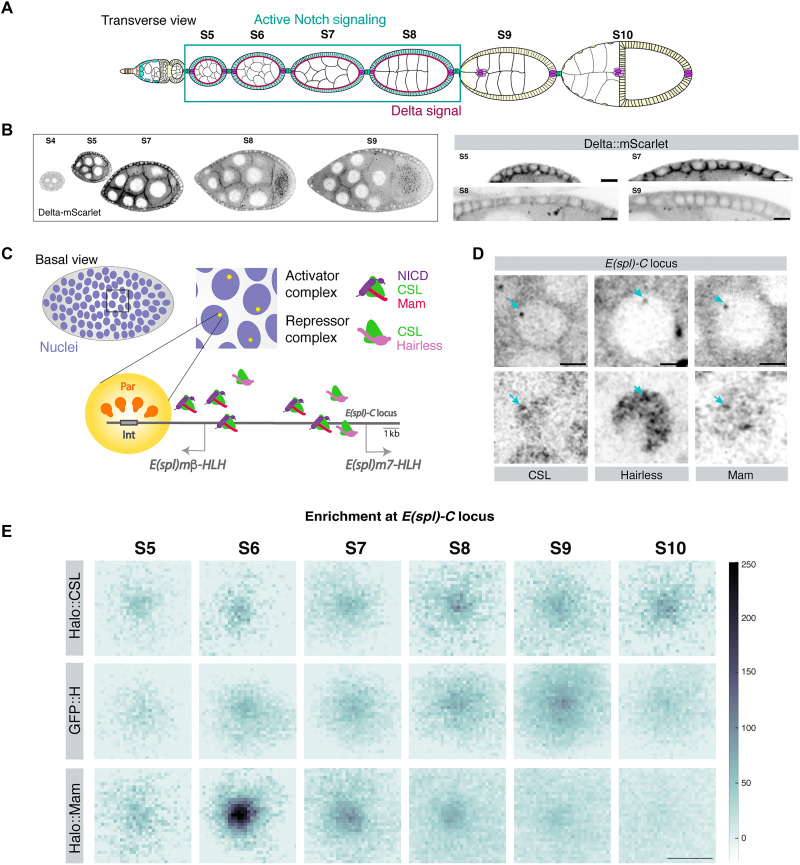
Mam hub forms at *E(spl)-C* during stages with endogenous Notch activity. (**A**) Cartoon of a *Drosophila* ovariole. The FE (pale blue and yellow) is a simple epithelium that surrounds the germ line (uncolored). Notch is active between stages 5 and 9 of development (blue rectangle) because of expression of Delta (magenta) on the surface of germline cells during those stages. (**B**) Confocal images from Delta::mScarlet showing expression between stages 4 and 9 (right) with higher-magnification images of the membranes at the FE-germline interface at the stages indicated. Scale bars represent 10 μm. (**C**) Diagram summarizing the method using Par/Int to label *E(spl)-C*. In follicle cell (FC) nuclei (purple), *E(spl)-C* is detected through binding of Par protein (yellow) to the *Int* sequence (gray) inserted within the endogenous locus. Recruitment at the locus of activator complexes [NICD (purple), CSL (green), and Mam (magenta)] or co-repressor complexes [CSL (green) and Hairless (light pink)] can be measured. (**D**) Live imaging of individual FC nuclei showing the location of the *E(spl)-C* locus (upper panels) and the localized enrichment of Halo::CSL, GFP::Hairless or Halo::Mam (lower panels). Scale bars represent 2 μm. (**E**) Average enrichment of the indicated proteins at *E(spl)-C* normalized to a random nuclear region (see Materials and Methods and fig. S1, A and B). CSL and Hairless are recruited in all stages, whereas Mam is enriched from stage 5 to 8, coincident with the Notch-active time window. The numbers of analyzed nuclei for Halo::CSL (221, 137, 377, 137, 259, and 158), Halo::Mam (92, 141, 173, 171, 287, and 219), and GFP::Hairless (94, 186, 241, 158, 248, and 207) were from at least three egg chambers (e.c.) per condition. The scale bar represents 1 μm.

The active Notch transcription complex, containing Mam, forms a local hub of enrichment at a target gene complex under conditions of prolonged ectopic activity (*15*). To investigate whether similar hubs occur when the pathway is endogenously active, we chose to focus on the follicular epithelium (FE) in the *Drosophila* ovary, which undergoes a period of Notch signaling between stages 5 and 8 of egg chamber development (*31*). Notch activity in this monolayer FE relies on ligands produced by the underlying germline cells (*32*, *33*), as illustrated by the expression of Delta::mScarlet, which is present in the membranes at the interface between the germ line and the follicle cells (Fig. 1, A and B). The broad distribution of Delta is consistent with the adjacent FE cells having an equivalent potential for Notch activity.

To investigate the formation of transcription hubs in this context, we analyzed the distribution of endogenously tagged factors by live imaging (*15*). The core transcription factor CSL and its partners, the coactivator Mam and the co-repressor Hairless, are all present in follicular cell nuclei throughout the period analyzed (fig. S1A). All exhibited a degree of heterogeneity within each nucleus, consistent with them forming dense foci or “hubs” (fig. S1B). To investigate whether these hubs are associated with Notch-regulated loci, we took advantage of the Par/Int system to identify the *E(spl)-C* locus in live tissues (*15*, *16*, *34*). This relies on the recruitment of fluorescently tagged Par binding proteins to their cognate *Int* sequences, which have been engineered into the *E(spl)-C* locus and result in a locus-specific fluorescent spot within each nucleus (Fig. 1, C and D). The levels of factors colocalizing with this tagged locus were then compared to those elsewhere in the nucleus. Results from multiple nuclei were pooled, and the average intensity centered on the locus region was visualized by heatmaps (Fig. 1E and fig. S1C).

All three members of Notch transcription complexes, CSL, Mam, and Hairless, were significantly enriched at the *E(spl)-C* locus during the stages analyzed (Fig. 1, D and E, and fig. S1D), whereas an unrelated transcription factor, Sox-box protein 14 (Sox14), was not (fig. S1E). The DNA-binding CSL was enriched from stages 5 to 10 and at broadly equivalent levels from stage 6 onward (Fig. 1E). In contrast, the coactivator Mam was enriched in a more restricted time window, from stage 5 to 8 with the highest levels detected during stage 6 (Fig. 1E). The period of Mam recruitment corresponds to the time when the Notch pathway is active (*31*, *35*), and the locus-specific enrichment of Mam was absent in tissues exposed to a γ-secretase inhibitor for 4 hours to block the activating cleavage (fig. S1F). These data demonstrate that Mam hub formation requires Notch activity. The average diameter of the Mam puncta is ~500 nm, which is close to the diffraction limit of the microscope, and the majority may be smaller on the basis of estimates from single-molecule tracking (*36*). Overall, they have similar size properties to the hubs formed by the Bicoid and Dorsal transcription factors in the embryo (*23*, *27*, *37*).

In the absence of Notch activity, CSL is associated with the co-repressor Hairless, and in agreement, Hairless was associated with the target locus during a more prolonged period and became more highly enriched in stages 9 and 10 (Fig. 1E). The fact that CSL and Hairless remain enriched after Notch is no longer active (stages 9 and 10) is consistent with a repressive role in those later stages and with evidence that CSL binding and chromatin accessibility persist for many hours after Notch activity and Mam recruitment cease (*15*). Furthermore, the presence of all three proteins during the period of Notch pathway activity fits with the proposal that dynamic exchange between activator and co-repressor complexes tunes the response to signaling (*9*, *15*). Here, we primarily focus on the notable recruitment of the co-activator Mam as a measure of a signaling-induced transcription hub at an endogenous locus.

### Dynamics of Notch target-gene transcription in follicular cells

**Fig. 2.**
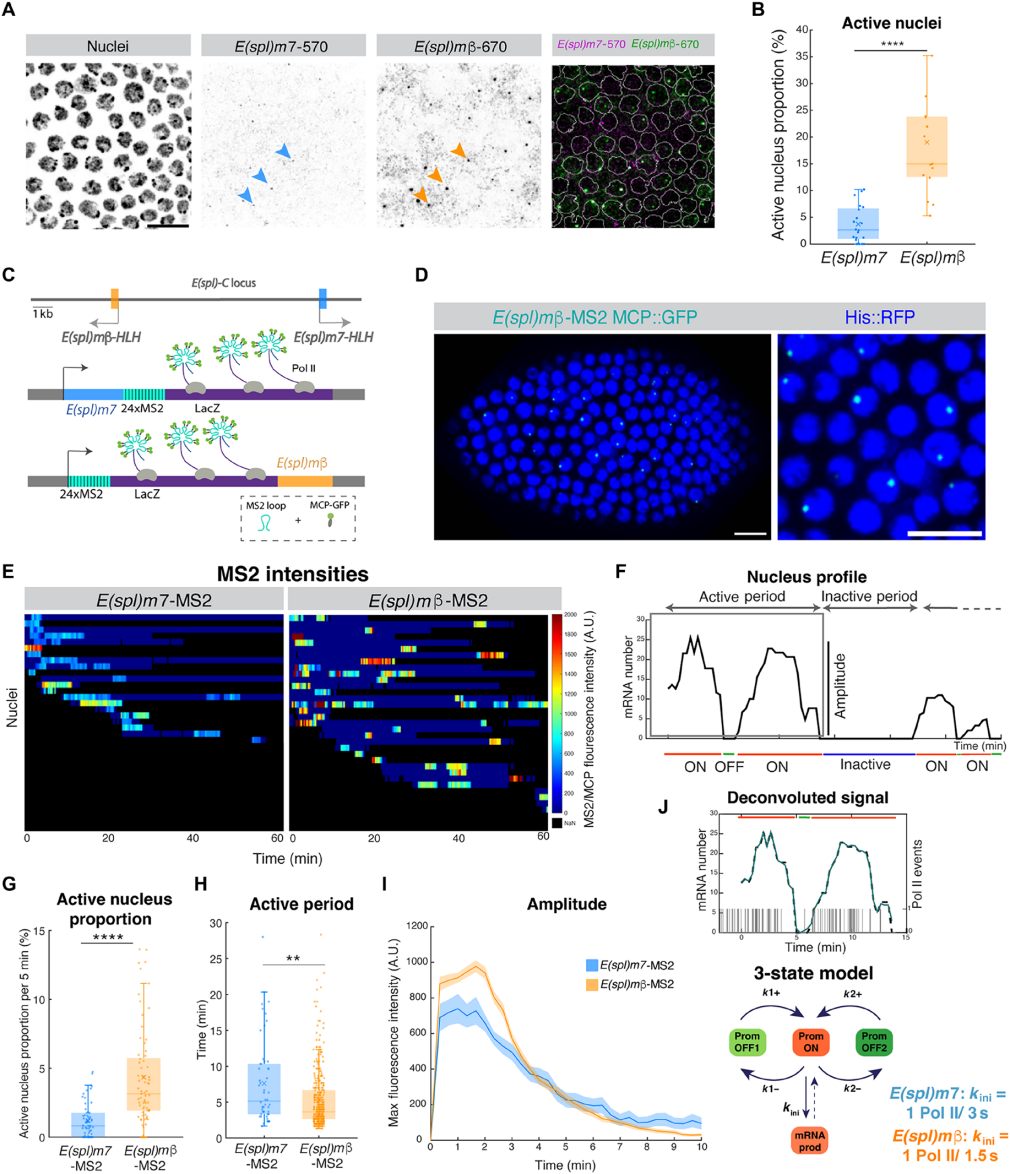
Transcription of Notch-responsive genes is probabilistic and pulsatile. (**A**) smFISH with *E(spl)m7*-570 and *E(spl)m*β-670 probes. ATSs for *E(spl)m7* (blue arrowheads) and *E(spl)m*β (orange arrowheads) colocalize. e.c. are stage 6 in this and subsequent figures. Scale bar, 10 μm. (**B**) Proportion of ATSs detected by smFISH. *n* = 20 (*m7*) and 18 (*m*β) e.c. (**C**) Diagram illustrating the MS2/MCP system. 24x MS2 loops (cyan) were introduced into *E(spl)m7 (blue)* and *E(spl)m*β (orange) coding regions with *LacZ* (purple) to extend RNA length. MCP-GFP (green) binds to MS2 loops in nascent RNA (Pol II; gray). (**D**) Image from the in vivo movie. *E(spl)m*β-MS2/MCP-GFP transcription puncta (cyan) are detected within nuclei (blue; H2Av-RFP). Scale bars, 10 μm. (**E**) Kymograph heatmap of maximum fluorescence intensity in active nuclei with time from *E(spl)m7*-MS2 and *E(spl)m*β-MS2 (movies S1 and S2). (**F**) Single transcription trace with active and inactive periods. Amplitude and bursting ON-OFF states are indicated. A.U., arbitrary units. (**G**) Average active nucleus proportion per 5-min period for *E(spl)m7*-MS2 (blue) and *E(spl)m*β-MS2 (orange). *P* = 3.16 × 10^−9^. (**H**) Active period durations for *E(spl)m7*-MS2 (blue) and *E(spl)m*β-MS2 (orange). *P* = 0.0018. (G and H) Box plots indicate the median, with 25 to 75 quartiles; error bars are SD. (**I**) Average intensities of *E(spl)m7*-MS2 and *E(spl)m*β-MS2 traces. Shading represents the SEM; *E(spl)m7*-MS2 (*n* = 66 nuclei) and *E(spl)m*β-MS2 (*n* = 443 nuclei) from six e.c. (**J**) Example MS2 intensity trace calibrated to mRNA numbers through time (black dashed line) with inferred trace (blue line) and corresponding Pol II initiation events (vertical lines) calculated using BurstDECOV (see Materials and Methods). At time 0, the MS2 spot is first detectable. Initial Pol II events precede MS2 detection because of transcription time. The three-state model, with one ON and two OFFs, is minimally required to explain data. *k*_ini_ differs between *E(spl)m7*-MS2 and *E(spl)m*β-MS2 (fig. S2, G to J). OFF state durations were calculated as 1/*k*1+ and 1/*k*2+ (*44*), with values in fig. S2J. **P < 0.01; ****P < 0.0001.

Live imaging indicates that Mam transcription complexes are enriched at *E(spl)-C* during the period of endogenous Notch activity. To investigate the relationship between the Mam hubs and target gene transcription, we began by characterizing the transcriptional response in the FE using single-molecule fluorescence in situ hybridization (smFISH) (*38*, *39*). Using fluorescently labeled probes for *E(spl)m7* and *E(spl)m*β, both of which are expressed in the FE in response to Notch signaling (*35*, *40*, *41*), we focused our analysis on the bright nuclear foci that correspond to the active transcription sites (ATSs) as a measure of the actively transcribing cells at each time point (Fig. 2A) (*38*). Only one ATS was detected per nucleus for each gene, and contrary to expectations, only a low proportion of nuclei contained an ATS for one or both genes at any given time point (Fig. 2B and fig. S2A). More nuclei contained ATSs for *E(spl)m*β (15 to 20% at stage 6) than for *E(spl)m7* (4 to 5% at stage 6) (Fig. 2B and fig. S2A). However, the majority (>90%) of *E(spl)m7* ATSs colocalized with an *E(spl)m*β ATS, suggesting that they respond to the same signaling input and that *E(spl)m*β has a higher probability of transcription than *E(spl)m7.*

Given the requirement for Notch activity throughout the epithelium and the duration of the developmental stage (*42*), all nuclei are likely to undergo one or more periods of transcriptional activity. The relatively low proportion of nuclei with an ATS at any time point suggests that each nucleus transcribes for short “active” periods, interspersed with longer periods of inactivity. To investigate this possibility and further analyze the dynamics of transcription at stage 6, we used the MS2/MCP system to image transcription from *E(spl)m7* and *E(spl)m*β in real time (*43*). MS2 loops were introduced into the endogenous genes by CRISPR-Cas9 engineering (see Materials and Methods) and the resulting strains combined with MCP::GFP and Histone::RFP so that transcribing nuclei could be identified and tracked in space and time (Fig. 2, C and D). Confocal live imaging of isolated ovaries revealed bright puncta in follicle cell nuclei corresponding to nascent transcription of the endogenous genes (Fig. 2, C and D, and movies S1 and S2). In both cases, transcription was highly asynchronous and stochastic between nuclei (Fig. 2E), as suggested by the distribution of ATSs and despite Delta expression in all neighboring cells and the requirement for Notch activity throughout the FE (*31*, *32*, *35*). Nevertheless, our data suggest that the transcriptional response is not purely stochastic but rather is promoted by specific conditions in the cellular signaling environment, as revealed by the coexpression of the two genes.

As illustrated by the kymographs (Fig. 2E), *E(spl)m7*-MS2 and *E(spl)m*β-MS2 transcription profiles were characterized by active periods of several minutes, during which transcription foci could be detected, followed by periods of inactivity (>7 min; Fig. 2, E and F). In agreement with the results from smFISH, only a low percentage of nuclei was active in any 5-min time window and a higher proportion of nuclei was transcribing *E(spl)m*β-MS2 than *E(spl)m7-*MS2 (Fig. 2G). The proportions are approximately half those detected by smFISH, a result that is consistent with only one of the two alleles being active at any time (given that only one allele has MS2 loops).

The two genes had active periods of similar durations, with those of *E(spl)m*β-MS2, on average, slightly shorter (Fig. 2H), and few nuclei underwent more than one active period within the ~1-hour time frame of imaging [6% for *E(spl)m7-*MS2 and 19% for *E(spl)m*β-MS2; fig. S2B], indicating that both genes can undergo inactive periods exceeding ~50 min. Even for those *E(spl)m*β-MS2 nuclei where two active periods were present, the majority was 10 min or longer (fig. S2C) and the lack of a second active period for most *E(spl)m7-*MS2 nuclei argues that it undergoes longer off-periods. Together, it is evident that the transcription profiles of both genes are highly dynamic with short periods of activity occurring at a relatively low frequency. However, once nuclei initiate transcription, maximal levels are achieved very rapidly (Fig. 2I), with *E(spl)m*β-MS2 reaching higher levels of mRNA production than *E(spl)m7-*MS2 (Fig. 2I). By calibrating accurately the MS2/MCP fluorescence against the measurements using smFISH (fig. S2, D to F), we estimate that a maximum of 35 RNAs are actively being transcribed at any time point during the height of *E(spl)m*β activity, with a detection threshold of three molecules.

Gene activity periods are usually composed of multiple ON and OFF cycles known as transcriptional bursts (*44*, *45*) brought about by rapid switching of the promoter. Consistent with this model, transcription levels of both genes fluctuated ON and OFF within each active period (Fig. 2, E and F). To investigate the promoter dynamics of the two genes during their respective active periods, we used a statistical inference method, BurstDECONV, to deconvolve the MS2/MCP signal traces into individual transcription initiation events (Fig. 2J and fig. S2G) (*44*, *45*). This generates a distribution of waiting times between successive polymerase initiation events, and the number of exponentials required to fit these data determines the number of promoter states in the model. As the resulting distributions for *E(spl)m*β and *E(spl)m7* active periods could not be fitted by a biexponential model (fig. S2H) but could be fitted with three exponentials, our analysis suggests the existence of three promoter states (Fig. 2J and fig. S2H) whose mean durations and probabilities were broadly similar for the two genes (fig. S2J). They comprise a competent promoter-ON state and two distinct promoter-OFF states, whose inferred durations were 140 to 150 s and 5 to 7 s, respectively. The longer OFF may correspond to a polymerase-paused state as seen with another developmentally regulated promoter (*44*). In the competent, promoter-ON state, the initiation rate *k*_ini_ is defined as the number of Pol II released per second. The main difference between *E(spl)m*β and *E(spl)m7* transcription kinetics was the magnitude of *k*_ini_, which was larger for *E(spl)m*β, consistent with the greater amplitude of its transcription intensity (Fig. 2, I and J, and fig. S2I). Thus, the two genes differ in the amount of Pol II released once a promoter is in the ON state, as well as in the probability that a nucleus will enter into an active period.

In summary, these results demonstrate that the transcriptional response to Notch activity in the epithelium is probabilistic. When active, nuclei transcribe for short periods and then enter a variable inactive period before reinitiating. This pattern is not simple to reconcile with the ensemble averaging for Mam enrichment at this stage and was unexpected in a tissue where the cells are exposed to a ligand over a prolonged period. However, the fact that both genes are active in the same nuclei [albeit fewer express *E*(*spl*)*m*7] argues that initiation of transcription is not purely stochastic but a response to specific cellular signaling conditions even if it differs from the highly penetrant and synchronized response in the mesectoderm (*46*).

### Mam hubs are dynamic and precede transcription initiation

**Fig. 3.**
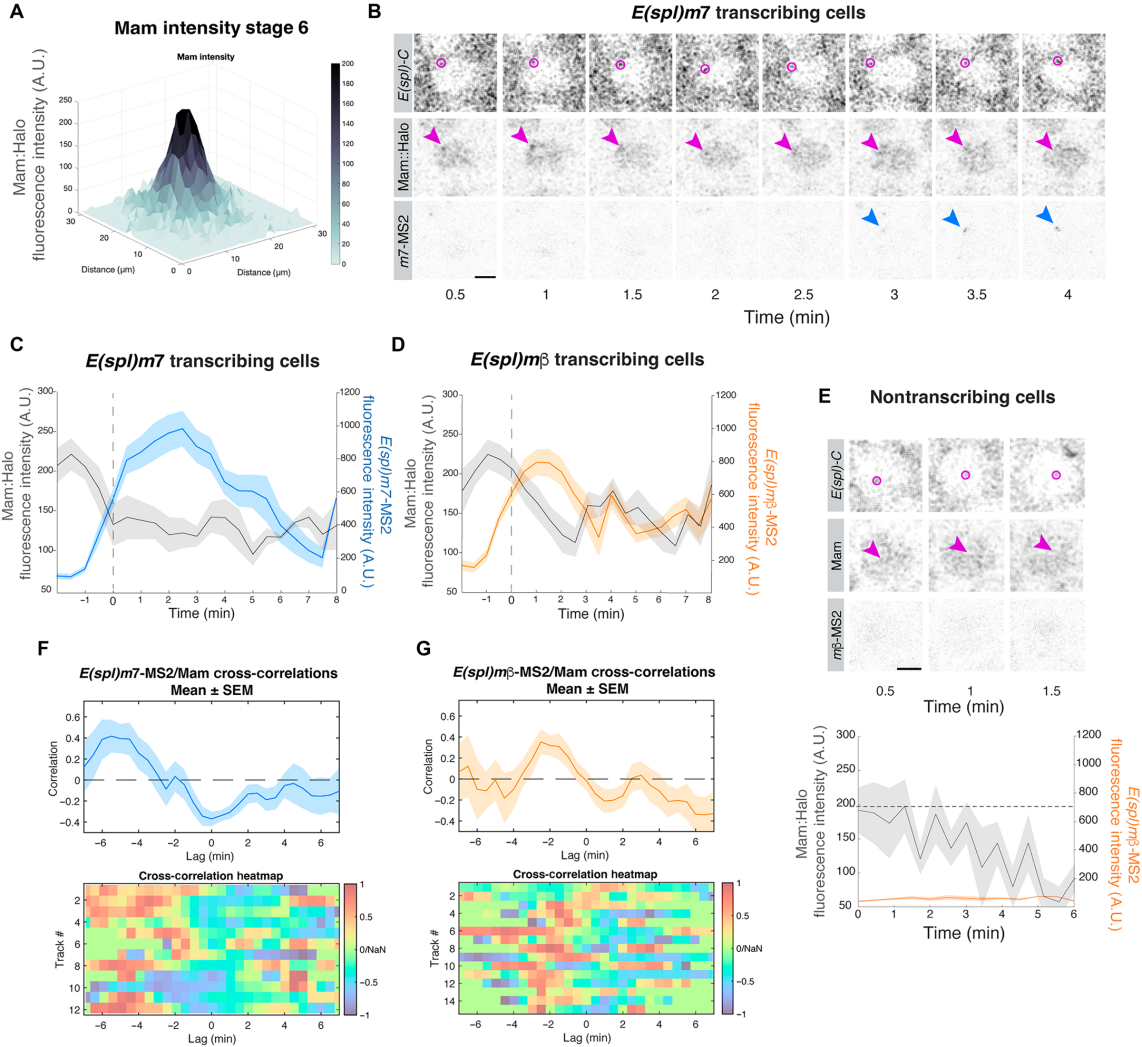
Mam hubs are dynamic and prefigure transcription. (**A**) Mean Mam intensity at *E(spl)-C* in stage 6, visualized by a 3D surface plot. *n* = 141 nuclei from three e.c. (**B**) Confocal images from the in vivo movie. Tracking Halo::Mam and *E(spl)m7*-MS2 in relation to the *E(spl)-C* locus (magenta circle). Mam enrichment (arrowhead) precedes *E(spl)m7*-MS2 transcription (blue arrowhead). The scale bar represents 2 μm (movie S3). (**C** and **D**) Average intensities of Mam (gray) and *E(spl)m7*-MS2 [(C), blue] or *E(spl)m*β-MS2 [(D), orange]. Intensities are time aligned with respect to transcription initiation (dotted line). *E(spl)m7-*MS2, *n* = 12 nuclei from seven e.c.; *E(spl)m*β-MS2, *n* = 15 from five e.c. (**E**) Confocal images from the in vivo movie of nontranscribing nuclei as in (B). No Mam enrichment occurs at *E(spl)-C.* Graph: Average intensities of Mam (gray) and *E(spl)m*β-MS2 (orange) in nontranscribing cells. Scale bars represent 2 μm. *n* = 7 nuclei from four e.c. (**F** and **G**) Cross-correlation analysis to test the relationship between transcription profiles and Mam enrichment. Mean correlation *R*^2^ value (top panels) and heatmaps (bottom panels) comparing the Mam profiles at different time lags with *E(spl)m7*-MS2 (F) and *E(spl)m*β-MS2 (G). In [(C), (D), (F), and (G)], shading represents the SEM.

Having observed that Mam is enriched at *E(spl)-C* (Figs. 1E and 3A) and that the transcriptional profiles are highly dynamic, we sought to investigate the relationship between the two. To achieve this, we performed live imaging in three channels, combining the ParB/Int–labeled *E(spl)-C* locus with Halo::Mam and *E(spl)m7-*MS2/MCP or *E(spl)m*β-MS2/MCP (Fig. 3B, fig. S3A, and movies S3 and S4). The levels of Mam at *E(spl)-C* were tracked for up to 3 min before transcription was first detected and throughout the active period. Aligning these Mam intensity levels with the onset of MCP/MS2 transcription revealed that there was a peak of Mam enrichment ~2 min before transcription onset, whichever gene was tracked (Fig. 3, C and D).

Mam enrichment at *E(spl)-C* was evident in all nuclei where transcription was initiated but did not reach levels above the nuclear average in nuclei that were not actively transcribing *E(spl)-C* genes, suggesting that a threshold mechanism might operate (Fig. 3E). These results support the hypothesis that formation of a Mam hub is a fundamental step in Notch-dependent activation of transcription but are difficult to reconcile with the cumulative Mam enrichment detected at this stage (Fig. 3A), given the relatively low frequency of active nuclei. We therefore returned to cumulative intensities of Mam at *E(spl)-C* and ranked the enrichments in quartiles (fig. S3B). This analysis revealed a gradation of enrichments, consistent with the dynamic fluctuations detected by live imaging.

The dynamic changes in Mam enrichment at *E(spl)-C* raise the possibility of a quantitative relationship between Notch activation complexes, as represented by Mam, and the transcriptional output. To assess this, we performed a cross-correlation analysis between the dynamic Mam and MCP/MS2 profiles for each nucleus (*27*, *47*, *48*). This tests the degree of similarity between two signals as a function of the time lag between them. Notably, there was a positive correlation between the Mam hubs and the transcription profiles for both genes, indicating that their rates of change are related to one another (Fig. 3, F and G). The correlation was maximal when the intensity profiles were shifted relative to one another by 2 min for *E(spl)m*β-MS2 and 5 min for *E(spl)m7-*MS2 (Fig. 3, F and G). This implies that the Mam hubs are maximal a few minutes before transcription is initiated. We also queried whether Mam enrichment levels correlated with the slope of the MS2/MCP transcription profiles, indicative of a direct relationship between Mam recruitment and Pol II loading. There was a positive correlation for *E(spl)m7*, arguing that the two are quantitatively related for this gene (fig. S3C). The lack of a similar correlation for *E(spl)m*β suggests that a response occurs once a threshold level of Mam is reached.

**Fig. 4.**
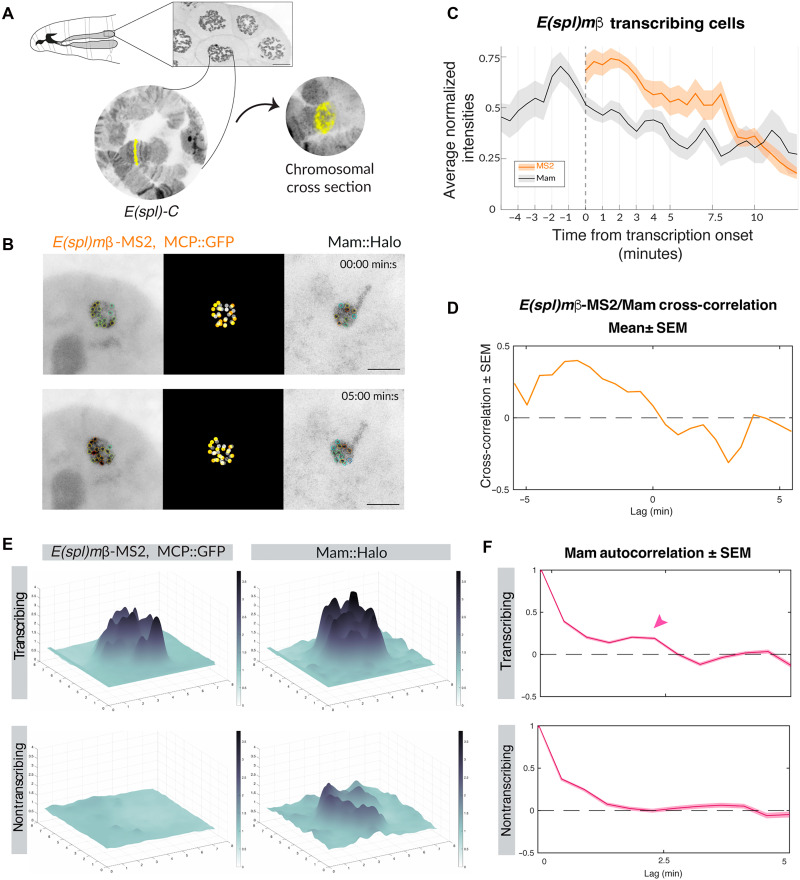
Condensed Mam hubs in salivary glands precede and correlate with transcription. (**A**) Cartoon illustrating the imaging approach in salivary glands. The upper image shows a small section of the tissue with nuclei. The scale bar represents 20 μm. Lower images show a zoomed-in view of *E(spl)-C* with the optical cross section revealing aligned chromosomal copies as separate foci as seen in (B). Yellow pseudocoloring signifies factors recruited to *E(spl)-C*. (**B**) Live imaging to track *E(spl)m*β-MS2 (grayscale, left; orange, middle) and Mam (gray, middle; grayscale, right) at *E(spl)*-C in cross sections of salivary gland chromosomes. Pairs of dense Mam foci and *E(spl)m*β-MS2 foci can be tracked over time (movie S5). Scale bars represent 5 μm. (**C**) Mean intensity profiles of Mam (gray) and *E(spl)m*β-MS2 (orange) aligned by transcription onset (dotted line, no quantification before onset). Shading represents the SEM. *n* = 20 tracks. (**D**) Cross-correlation analysis to test the relationship between paired *E(spl)m*β-MS2 transcription profiles and Mam enrichment profiles, with the mean correlation *R*^2^ value at different time lags. For heatmaps, see fig. S4C. (**E**) 3D plot of *E(spl)m*β*-*MS2 and Halo::Mam enrichment at *E(spl)-C* in transcribing (upper panels) and nontranscribing cells (lower panels) at a single time point from movie S6. (**F**) Autocorrelation analysis to test for periodicity in Mam enrichment profiles. Autocorrelation values in transcribing (upper panels) and nontranscribing cells (lower panels) reveal a second peak (arrow) in transcribing nuclei.

We have previously detected Notch-dependent Mam enrichments associated with *E(spl)-C* in another tissue, the salivary glands (*15*). Under those conditions, an additional signal, provided by the hormone ecdysone, was required to bring about robust transcription. To investigate whether the dynamics between the Mam hub and transcription are conserved in this tissue, we imaged the *E(spl)-C* locus in Notch-ON, ecdysone-treated nuclei in combination with *E(spl)m*β-MS2/MCP at a high resolution (Fig. 4A). This tissue is unusual in having polytene chromosomes containing many aligned copies of the genome. When imaged at a high resolution in a cross section, we could detect heterogeneities within the large diffuse “nuage” of Mam recruited around *E(spl)-C* (Fig. 4B and movie S5). The punctate high-density Mam regions, which we refer to as dense hubs, were associated with *E(spl)m*β-MS2 signals such that most Mam dense hubs were paired with MS2 foci and vice versa (Fig. 4B, fig. S4A, and movie S5).

Tracking the paired Mam and *E(spl)m*β-MS2 foci, we detected a peak of maximal Mam enrichment just before transcription started (Fig. 4C). Comparing quantitatively the profiles of Mam in the dense hubs with the transcription intensity profiles, by performing a cross-correlation analysis on each pair, confirmed that the two are positively correlated (Fig. 4D and fig. S4C). As with Mam hubs in the FE, the maximum correlation was achieved when the two profiles were aligned with a lag, indicating that the Mam hub achieves maximal levels shortly before transcription is initiated and that the rates of change are related (Fig. 4D). To assess Mam behavior in nuclei that were not transcribing, we imaged tissues without ecdysone treatment. These nontranscribing nuclei had a more homogeneous diffuse “nuage” of Mam enrichment across the chromosome, as described previously, and lacked the dense foci of transcribing nuclei (Fig. 4E, fig. S4B, movie S6) (*15*). On this basis, we propose that the smaller dense foci are analogous to those found in the FE. In both cases, they are present transiently and achieve maximal levels before transcription. In addition, a correlation analysis of the Mam profiles against themselves (autocorrelation; Fig. 4F) revealed a small positive correlation at ~2.5 min, suggesting that there is a periodicity in the dynamics of hub condensation under these conditions of ectopic Notch activity.

Overall, the results demonstrate that Mam becomes dynamically enriched into a dense hub that precedes and correlates with transcription being initiated at the Notch-regulated genes. We propose that this is a core feature of the mechanism for initiating transcription downstream of Notch signaling.

### The Mam hub is stabilized when transcription is inhibited

**Fig. 5.**
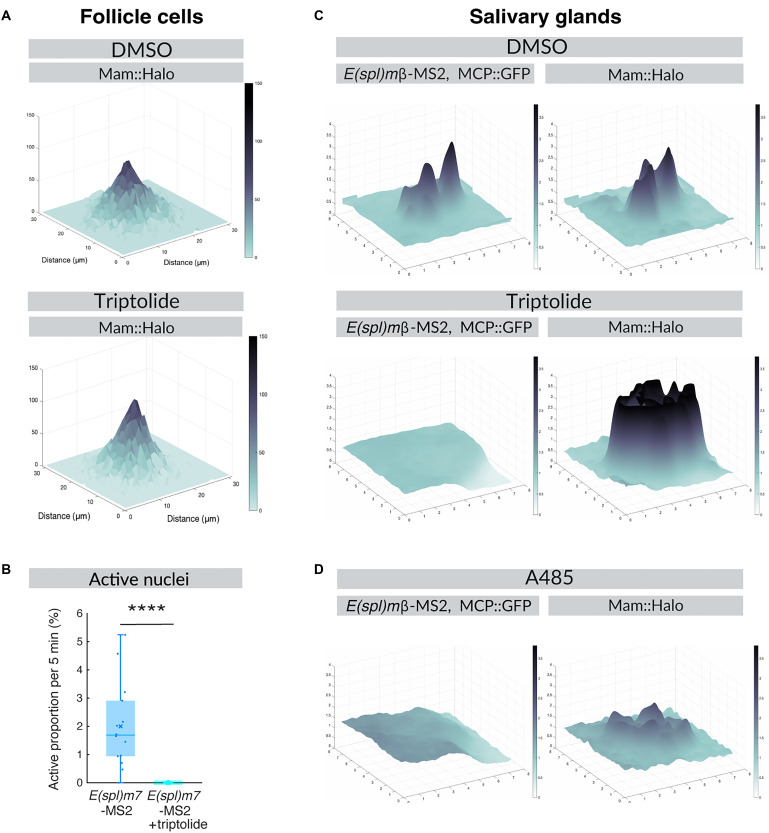
Mam hub persists when transcription is inhibited. (**A** and **B**) Effect of triptolide treatment on Mam enrichment and transcription in follicle cells. (A) 3D surface plot of average Mam intensity levels at *E(spl)-C* in control [dimethyl sulfoxide (DMSO)] or triptolide-treated tissues. *n* = 531 and 540 from seven and six e.c. (B) Average proportions of *E(spl)m7*-MS2 transcribing nuclei from stage 6 e.c. before and after triptolide treatment (*n* = 3). (**C**) Effect of triptolide treatment on Mam enrichment and transcription in salivary glands. 3D surface plot of *E(spl)m*β-MS2/MCP and Mam intensity levels at the *E(spl)-C* locus in control (DMSO) or triptolide-treated tissues at a representative time point. (**D**) Effect of A485 treatment on Mam enrichment and transcription in salivary glands. 3D surface plot of *E(spl)m*β-MS2/MCP and Mam intensity levels at the *E(spl)-C* locus in control (DMSO) or A485-treated tissues at a representative time point. ****P < 0.0001.

Mam enrichment into a local hub is related to transcriptional activity. To distinguish whether it is a cause or a consequence of transcription being initiated, we tested the consequence of pretreating the tissues with the highly specific transcription inhibitor triptolide, which impedes preinitiation complex formation (Fig. 5, A and B) (*49*). Live imaging of *E(spl)m7*-MS2/MCP after tissues were treated with triptolide for 1 hour confirmed that transcription had been completely abolished (Fig. 5B). Notably, however, Mam levels at *E(spl)-C* were enhanced under these conditions, giving rise to a more intense and compact Mam hub (Fig. 5A). A second transcription inhibitor, 5,6-dichloro-1-β-d-ribofuranosylbenzimidazole (DRB), which interferes with the release of paused Pol II, also brought about a similar increase in the intensity of the Mam hubs (fig. S5, A and B). These results demonstrate that transcription per se is not required for the recruitment of Mam into a hub. They suggest the converse that transcription/mRNA production destabilizes the hub and potentially contributes to its dissolution.

Comparable results were obtained in the salivary glands. Although there was little change in the diffuse Mam recruitment in nontranscribing nuclei following triptolide treatment (*15*), we detected a substantial increase in Mam intensities within dense hubs of transcriptionally active nuclei, as in the FE (Fig. 5C). As Mam is reported to interact with the histone acetylase CBP/p300 (*13*, *14*, *50*), we also analyzed the consequences of exposing tissues to A485, a potent inhibitor of its acetylase activity. CBP/p300 inhibition resulted in a substantial decrease in the fluorescence intensity of the dense Mam hubs (Fig. 5D). A485 treatment did not fully eliminate the dense puncta, and the surrounding “nuage” was not altered (Fig. 5D) (*15*), suggesting that other factors also contribute to robust Mam recruitment. However, these data reveal that Mam condensation is slowed or less efficient in the presence of A485, implying that CBP/p300 normally plays an important role similar to its positive effects on Sox2 recruitment (*51*–*53*).

Together, these data argue first that the formation of dense Mam hubs at *E(spl)-C* is independent of transcription and is enhanced by the activity of CBP/p300. Second, it appears that the Mam foci are normally destabilized when transcription progresses. This would generate a cyclical loop of Mam condensation and Mam dispersal in response to signaling (*54*).

### The Mam hub is sensitive to Notch activity levels

Mam is a core component of the activation complex, recruited by NICD. We therefore hypothesized that the intensity of Mam hubs would be directed by the levels of Notch activity. To address this question, we used genetic strategies to increase or reduce Notch and monitored the consequences on Mam recruitment and transcription during stage 6.

**Fig. 6.**
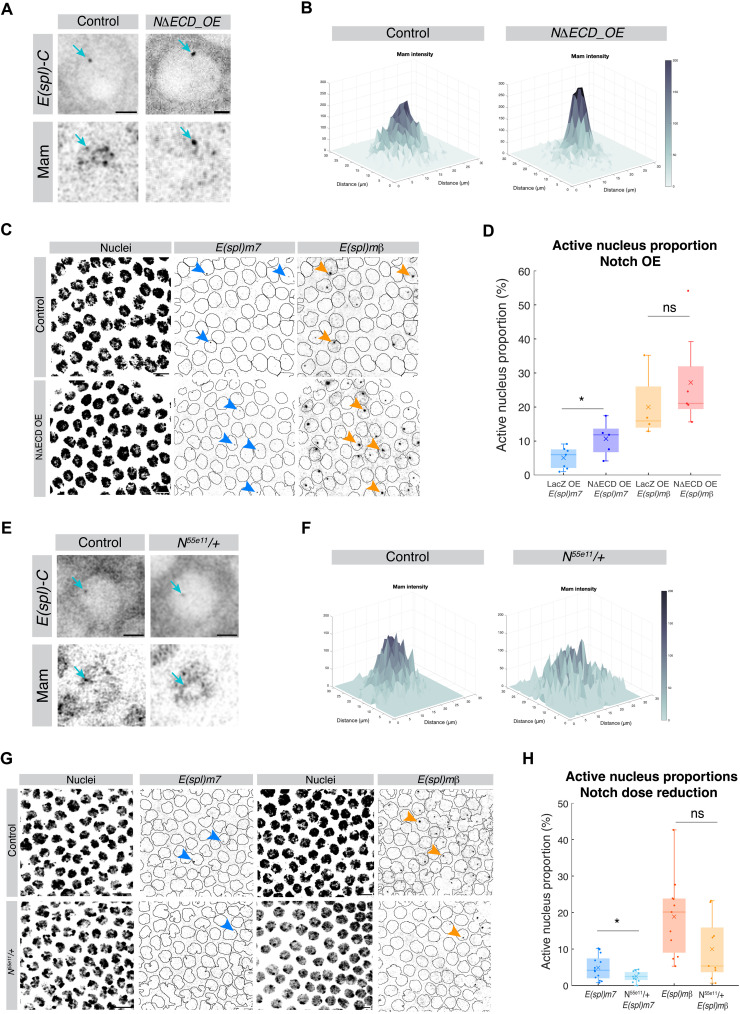
Effects of Notch activity levels on Mam enrichment and transcription profiles. (**A** to **D**) Elevated Notch activity. (A) Live imaging of *E(spl)-C* (upper panels, cyan arrows) and Mam hubs (lower panels, cyan arrows) under control (*UAS-LacZ*) (left) and Notch overexpression conditions (*UAS-N*Δ*ECD*) (right) (lower panels, cyan arrows). Scale bars represent 2 μm. (B) 3D surface plot of mean Mam intensity at *E(spl)-C* under control (left) and NΔECD overexpression (right) conditions. *n* = 240 (*UAS-LacZ*) and *n* = 279 (*UAS-N*Δ*ECD*). (C) Confocal images of smFISH with *E(spl)m7*-570 and *E(spl)m*β-670 probes in control (*UAS-LacZ*; upper panels) or increased *Notch* (*UAS-N*Δ*ECD*; lower panels). DAPI labels nuclei (left panels), generating masks to score ATSs (right panels). Scale bars, 10 μm. (D) Proportions of active nuclei in control and NΔECD detected by smFISH for both target genes. Mean data for e.c.; *n* = 4 to 6. (**E** to **H**) Reduced Notch activity. (E) Live imaging of *E(spl)-C* (upper panels, cyan arrows) and Mam hubs (lower panels, cyan arrows) under control (left) and Notch heterozygous conditions (*N^55e11^/+*) (right) (lower panels, cyan arrows). Scale bars represent 2 μm. (F) 3D surface plots of Mam mean intensity at *E(spl)-C* under control (left) and Notch heterozygous (right) conditions. *n* = 137 (control) and 76 (*N^55e11^/+*). (G) Confocal images of smFISH with *E(spl)m7*-570 and *E(spl)m*β-670 probes in control (upper) or reduced Notch (*N^55e11^/+*; lower panels). DAPI labels nuclei used to form mask outlines. Scale bars, 10 μm. (H) Proportions of active nuclei in control and *N^55e11^/+* detected by smFISH for both target genes. Mean data for e.c., *n* = 9 to 15. ns, not significant. *P < 0.05.

To investigate the consequences from increasing Notch activity, a constitutively active form of Notch, Notch∆ECD, was expressed at moderate levels throughout the follicular epithelium. Under these conditions, Mam enrichment levels at *E(spl)-C* were strongly elevated, giving rise to a dense hub in many nuclei and less diffuse nucleoplasmic protein (Fig. 6, A and B). The effects of elevated Notch on transcription were then analyzed by smFISH and by tracking transcription live with MS2/MCP. In the latter case, genetic constraints resulted in conditions with a lower level of Notch∆ECD being present (see Materials and Methods). Two main consequences from elevated Notch were seen. First, there was an increase in the proportion of actively transcribing nuclei (Fig. 6, C and D, and fig. S6A). This was most evident for *E(spl)m7*, which showed a marked increase using both approaches, while for *E(spl)m*β, the difference was only evident under higher overexpression conditions, as seen by smFISH, where the proportion and size of the ATSs were both increased (Fig. 6, C and D). Second, the amplitude of transcription, measured by *E(spl)m7*-MS2 and *E(spl)m*β-MS2 transcription profile intensities, was increased for both genes (fig. S6D), while the active and inactive period durations were unaffected (fig. S6, B and C). Thus, the elevated Notch activity results in a greater probability of nuclei entering an active period and a higher level of mRNA production within each active period, which correlates with the increase in Mam hubs.

To reduce Notch levels, we generated tissues that were heterozygous for a loss-of-function Notch allele (*N^55e11^*). Under these conditions, the enrichment of Mam at *E(spl)-C* was decreased (Fig. 6, E and F), and the proportion of nuclei actively transcribing *E(spl)m7* and *E(spl)m*β, on the basis of the ATS analysis, was reduced (Fig. 6, G and H). Likewise, a lower fraction of nuclei was transcribing *E(spl)m7*-MS2 (fig. S6E). The proportions transcribing *E(spl)m*β-MS2 were highly variable, with no consistent reduction, suggesting that the residual Notch is close to the threshold required for its activation (fig. S6E). The amplitude of transcription during the active period and the active/inactive period durations were unaffected (fig. S6, F to H).

Together, these observations demonstrate that Mam recruitment is sensitive to altered Notch activity levels in a manner that allies with the consequences on gene transcription, primarily with the probability of a nucleus actively transcribing and, to a lesser extent, with the levels of mRNA then produced.

## DISCUSSION

**Fig. 7.**
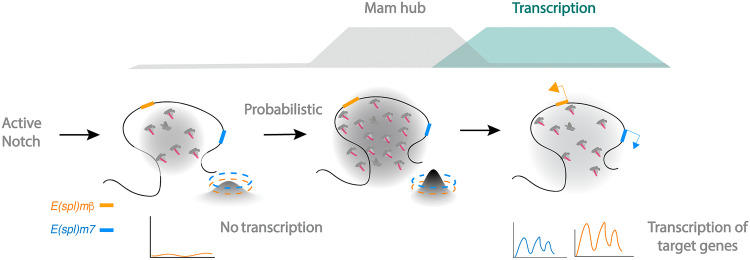
Model illustrating the dynamic relationship between Mam hubs and transcription. Mam-containing transcription complexes (magenta symbols) assemble in response to Notch activation and are recruited to *E(spl)-C* containing *E(spl)m7* (blue) and *E(spl)m*β (orange). (Left) Low levels of Mam recruitment in most nuclei (gray shading and 3D plot) are below the threshold required to promote transcription of *E(spl)m7* (3D plot, blue dash) or *E(spl)m*β (3D plot, orange dash). In a proportion of nuclei (middle), a more concentrated Mam hub is formed (gray shading) because of higher Notch levels and is sufficient to promote transcription (green shading) of one or both genes according to their different thresholds (3D plot, orange/blue dash). The concentration of Mam precedes and correlates with transcription (top curves). Once transcription has initiated, the density of Mam at *E(spl)-C* is dispersed (right), and the cycle can be reinitiated.

Transcription factor hubs or condensates have been observed in several different biological contexts and organisms, and there is growing evidence of a functional link with transcriptional regulation (*25*, *27*, *55*). However, their role in endogenous cellular processes remains enigmatic because of the challenge from probing their existence in physiological contexts. Here, by live imaging of the co-activator Mam in tissues with endogenous Notch signaling, we demonstrate that high-density Mam enrichments, which we refer to as dense hubs, are induced under conditions with active signaling and that their formation precedes and correlates with gene activity, specifically with the probability and levels of transcription (Fig. 7). These mature transcription-competent hubs are the final step linking Mam recruitment to promoter activation, and their stepwise assembly may be a common feature of transcription factor hubs and condensates, as observed for the maturation of transcription hubs seeded by the pioneer factor Zelda (*15*, *56*). Understanding how cells decode signals and generate accurate transcription outcomes is of fundamental importance for development, adult homeostasis, and disease. Our data implicating a key role for transcription hubs in implementing the Notch signal shed light on how activity levels inform the amount of mRNA transcribed from a target gene.

The characteristics of the Mam-enriched dense hubs are similar to those observed for synthetic (Gal4-VP16) and endogenous transcription factors in early *Drosophila* embryos (*25*, *27*) and in other contexts (*57*–*59*). As in those examples, localized Mam enrichments of less than 500 nm are present shortly before transcription onset and decrease in intensity as transcription progresses. Visualizing the interplay between hubs and nascent transcription in single cells at a high spatiotemporal resolution made it possible to uncover this relationship, which would be masked by ensemble averaging. The correlations between the hub intensities and the active transcription periods suggest that high-density Mam hubs directly coordinate transcription initiation, likely through the recruitment or release of RNA Pol II (*15*, *57*, *60*). The subsequent decrease in hub intensity is similar to that observed for Zelda hubs and also fits with predictions from the “kiss and kick” model, in which enhancer promoter contacts are transient (*61*). The decrease may be a direct consequence of nascent RNA production, which has been shown to destabilize transcription factor condensates (*62*). In agreement, the increased intensity/stability of Mam hubs we detect with triptolide or DRB treatment argues that their dynamics are regulated by the transcription cycle, as observed for Mediator condensates (*63*, *64*). As degradation of NICD has also been linked to the transcription cycle, this may also contribute to the dynamics of Mam recruitment (*13*, *65*). In addition, CBP/p300 acetylase activity appears to promote the maturation into dense Mam hubs, similar to its roles in the assembly of other transcription hubs (*55*, *66*). Whichever the mechanisms, the temporal properties of Mam hub formation and dissolution provide a vehicle for transcriptional decoding of dynamic signaling inputs (*54*).

Given that Mam requires NICD for assembly into the transcription complex with CSL, we hypothesized that dynamic changes in Notch signaling would translate into Mam levels within the transcription hub. In agreement, locus-specific Mam enrichment was detected during stages when transcription of Notch-responsive targets such as *E(spl)m7* and *E(spl)m*β occurs and was correlated with the ensuing profiles of transcription. Furthermore, changes in levels of Notch activity altered the amount of Mam within the hub in a manner related to the probability and amplitude of transcription response. In the multicopy *Drosophila* polytene chromosomes, where we previously detected a diffuse Notch signaling–induced hub in the absence of transcription (*15*), a change in the local distribution of Mam heralds the transition to transcriptionally active nuclei when it becomes concentrated into dense foci that prefigure and cross-correlate with sites of active transcription in a similar manner. All of these characteristics support the model that activator-coactivator hubs form in response to signaling and confer a probability of transcription that relates to their intensity and durations (Fig. 7).

As endogenous Notch signaling is maintained over a prolonged period in the FE, we were initially surprised to find that transcription of *E(spl)m*β and *E(spl)m7* occurred sporadically. Despite this, their ATSs coincide, demonstrating that firing is not random and suggesting that their probability of transcribing is related to fluctuating levels of Notch activity, as seen for Notch-regulated genes in *Caenorhabditis elegans* gonads (*67*). In agreement, both *E(spl)m*β and *E(spl)m7* had a higher probability of transcribing when the Notch activity levels were mildly increased. Of the two genes, *E(spl)m7* had a lower probability and amplitude of transcription, a pattern that is also evident in single-cell RNA data from this tissue (*40*, *41*), suggesting that *E(spl)m7* requires a higher Notch threshold than *E(spl)m*β, at least in this tissue (Fig. 7). Concordantly, *E(spl)m7* transcription profiles were more sensitive to changes in Notch activity.

The differing profiles also imply that a Notch-induced Mam hub of a given size/density yields different outcomes depending on the promoter affected. In a similar manner, different target genes of the Dorsal transcription factor showed distinct requirements with respect to the properties of the Dorsal hub (*27*). Explanations likely lie in sequences present in the enhancers/promoters that will recruit additional factors. For example, these might prime *E(spl)m*β so that it is poised for activation or repress *E(spl)m7* to confer a more refractory state (*46*, *68*). As a result, one gene is more frequently activated than another under conditions where Notch activity levels are close to the threshold. This type of probabilistic behavior may be common not only for Notch-regulated genes but also much more widely in signaling-induced responses as many genes have demonstrated distinct discontinuous transcriptional activities that result in high degrees of cellular heterogeneities.

## MATERIALS AND METHODS

### Experimental animals

The species used was *Drosophila melanogaster*. Flies were grown and maintained on food consisting of the following ingredients: glucose (76 g/liter), cornmeal flour (69 g/liter), yeast (15 g/liter), agar (4.5 g/liter), and methylparaben (2.5 ml/liter).

### Fly stocks

Full genotypes are summarized in table S1. To image the *E(spl)-C* locus, a recombinant “locus tag” chromosome (*16*) consisting of *Int1* sequences inserted into the *E(spl)-C* intergenic region adjacent to *E(spl)mdelta* (chromosome 3R 26038865:26038884) and *UAS-ParB1-mcherry*– or *UAS-ParB1-GFP*–inserted *AttP.86Fb* was used in combination with *traffic-jam-Gal4* (*tj-Gal4*) to drive ParB1 expression in the FE (*69*) or with *1151-Gal4* to drive expression in salivary glands (*16*). To analyze the expression and enrichment of TFs and other proteins, we used endogenously tagged fluorescently labeled proteins generated by CRISPR, Halo::Mam, GFP::Mam (*15*), Sox14::GFP (BL-55842), and Dl::mScarlet (*70*) or by genomic rescue constructs Halo::CSL (*15*), GFP::CSL, and GFP::Hairless (*16*).

For MCP/MS2 live imaging of transcription, 24 MS2 loops and the lacZ coding sequences were inserted into *E(spl*)*m7-HLH* and *E(spl*)*m*β-*HLH* genes by CRISPR-Cas9 genetic engineering. In *E(spl*)*m7*-MS2, the insertion sire was close to the C terminus of the coding sequences using the strategy described (*46*), and in *E(spl*)*m*β-MS2, the insertion site was closer to the transcription start site (*15*, *70*). The MS2-tagged genes were combined with *hsp83-MCP::GFP* (BL-7280) to track transcription and His2Av-RFP (BDSC no. 23650) to label nuclei. *hsp83-MCP::GFP* (BL-7280) was also recombined with *tj-Gal4* to generate a strain for triple labeling experiments with Halo::Mam, *E(spl)-C* “locus tag,” and the *E(spl*)*m7*-MS2 or *E(spl*)*m*β-MS2 chromosome. For similar experiments in the larval salivary glands, flies were generated containing *1151-Gal4*, *hsp83-MCP::GFP*, *E(spl)m*β-MS2, Halo::Mam, and *UAS-N*∆*ECD*.

Notch overexpression was achieved either by combining *tj-Gal4::tub-Gal80*^*ts*^ with *UAS-N∆ECD* (*15*), generating analogous stocks with *UAS-LacZ* as a control, or in experiments with MS2/MCP, by combining the same UAS lines with a “leaky” Flip-out cassette (*Tub>FRT.STOP.FRT>Gal4::UAS-TandemTomato*). Crosses were maintained at 18°C (Notch at endogenous levels) or 29°C for 24 hours (Notch overexpression). For Notch dose reduction, *N*^*55e11*^*FRT19A* was combined with the relevant Halo::Mam and MS2/MCP, and *yw* was used as a control.

### Method details

#### Tissue preparation, drug treatments, and live imaging conditions

Ovaries were dissected and mounted in Schneider’s medium (Sigma-Aldrich/Merck, S0146) supplemented with 15% (v/v) fetal bovine serum (Sigma-Aldrich, F2442), 0.6% (v/v) streptomycin/penicillin antibiotic mix (Invitrogen, 15140-122), and insulin (0.20 mg/ml; Sigma-Aldrich, 15500). For imaging, ovaries were mounted into 35-mm poly-d-lysine–coated glass-bottom dishes (Mattek, P35GC-1.5-10-C). For drug treatments, dissected ovaries were incubated with compound E (4 hours, 100 nM; Abcam, HY-14176), DRB (1 hour, 500 μM; Santa Cruz Biotechnology, 53-85-0), or triptolide (1 hour, 10 μM; Sigma-Aldrich, T3652) before imaging. For experiments testing whether transcription was abolished, triptolide was added at 10 μM after imaging was initiated. Preparation of salivary glands was as described previously (*15*, *16*).

Live confocal fluorescence imaging of ovaries was performed on a Leica SP8 microscope equipped with seven laser lines (405, 458, 488, 496, 514, 561, and 633) and using a 40× apochromatic 1.3 oil immersion objective and two hybrid GaAsP detectors. For Mam enrichment assays, individual egg chambers were imaged with a 4× zoom, 1024-by-1024 pixel resolution, pinhole set to 1.5 Airy, three line averages, a 12-bit depth, and a 400-Hz scanning speed. *Z*-Stacks were 0.5 μm wide, and a total of ~30 stacks were acquired to cover most of the epithelium at the imaged surface of the egg chamber. For MS2/MCP movies, individual egg chambers were imaged at a 3.5× zoom, 512-by-512 pixel resolution, pinhole set to 3.5 Airy, an 8-bit depth, and a 400-Hz scanning speed. *Z*-Stacks of 0.5 μm in width for 30 stacks were acquired. The time frame was 20 s. The same settings for MCP-GFP detection were used: 29.36-mW, 488-nm argon laser detected with a hybrid detector.

For Mam enrichment together with MS2/MCP movies, egg chambers were imaged using the ×63/1.4–numerical aperture HC PL APO CS2 oil immersion objective at a 3.5× zoom, 512-by-512 pixel resolution, pinhole set to 1.5 Airy, a 12-bit depth, and a 600-Hz scanning speed. *Z*-Stacks were 0.5 μm wide, and ~15 stacks were acquired. The time frame was 30 s. Similar conditions were used for salivary glands, except a 10× zoom and pinhole set to 2-Airy.

#### Single-molecule in situ hybridization

Custom smFISH probe sets for *E(spl)m7-HLH* and *E(spl*)*m*β-HLH genes were designed with Stellaris Probe Designer (Bioscience Technologies). Ovaries were dissected in nonsupplemented Schneider’s medium and fixed for 30 min in 3.7% formaldehyde:phosphate-buffered saline (PBS) at room temperature. Ovaries were then washed three times in PBST (PBS + 0.1% Triton X-100) and transferred to methanol stepwise with 5-min incubations in PBST:MeOH (7:3), PBST:MeOH (1:1), and PBST:MeOH (3:7) on a nutating mixer before transferring to 100% MeOH. After 10 min in 100% MeOH, tissues were rehydrated with the same solutions in the reverse order (3:7 PBST:MeOH, 1:1 PBST:MeOH, and 7:3 PBST:MeOH). After rinsing with PBST, ovaries were prehybridized with WB-A (Biosearch Technologies, SMF-HB1-10) for 10 min and then transferred to a hybridization mixture prepared by adding probes to 100 μl of RNA FISH Hybridization Buffer (Biosearch Technologies, SMF-WB1-60) at 1:100% (v/v) from a 12.5 μM stock. They were then incubated at 37°C for 16 to 18 hours in the dark, washed twice for 30 min with prewarmed WB-A, rinsed with PBST, and then mounted using Vectashield with 4′,6-diamidino-2-phenylindole (DAPI).

smFISH samples were imaged using a Leica SP8 microscope equipped with different laser lines (405, 458, 488, 496, 514, 561, 594, and 633) and a 40× apochromatic 1.3 oil immersion objective, and one hybrid GaAsP detector was used. Individual egg chambers were imaged with a 4× zoom, 1024-by-1024 pixel resolution, pinhole set to 1 Airy, three line averages, a 12-bit depth, and a 400-Hz scanning speed. *Z*-Stacks were 0.5 μm in width with a total of around 30 stacks.

### Analysis

#### Image analysis pipelines

To measure enrichments, images were analyzed using MATLAB by importing Leica Images with the BioFormat package (MATLAB R2021b, MathWorks and openmicroscopy.org). Using a custom MATLAB app, a squared region of interest (ROI), 30 by 30 pixels centered on *E(spl)-C*, was selected [adapted from (*15*)]. Fluorescence levels of Halo::Mam and other factors were then measured within this ROI, averaged per experimental condition and normalized with an average value, and calculated from ROIs placed at random in the same set of nuclei. Average pixel plots and profile graphs were generated, ROIs were averaged in the *y* dimension, and the mean and SEM were represented for each condition.

For quantification of Mam enrichment together with MS2 movies, *E(spl)-C* was tracked with TrackMate, the resulting ROI was assigned to the Mam and MS2 channels, and fluorescence intensities were obtained using a custom MATLAB script. Both Mam and MS2 signals were normalized by subtracting the background level (minimum value from the trace) and median filtered to smooth the traces.

For smFISH images, nuclei were segmented in two dimensions using Weka Segmentation (Fiji plug-in) to detect Hoechst staining, masks were generated using watershed after transformation to a binary image, and their number indicated the total number of nuclei analyzed per egg chamber. Masks were applied to the fluorescent probe channels to eliminate the signal coming from the cytoplasm, and a minimal threshold was used to identify the prominent ATS. The Fiji plug-in Analyze Particles was used to count the total number of ATSs in an image, and totals were divided by the number of nuclear masks and represented as the proportion of active nuclei.

For MS2/MCP movies, the His2Av-RFP or siRDNA (Spirochrome SC007) signal was used to segment and track the nuclei in two dimensions. Each movie was segmented using Weka Segmentation (Fiji plug-in) and watershed after transformation to a binary image to obtain masks. Tracking of the nuclei was performed using TrackMate (Fiji plug-in), detecting the nuclei as masks and allowing to track a maximum distance of 3 μm, a maximum gap distance allowed of 3 μm, and a maximum frame gap of two frames. A custom MATLAB script (MATLAB R2021b, MathWorks), adapted from (*46*), was used to detect and track the transcription spots on the basis of the three-dimensional (3D) Gaussian method developed in (*43*). The tracked transcription spots were overlapped with the ROIs (masks) of the tracked nuclei, and spots lying outside nuclei were removed from the analysis. We note that under Notch overexpression conditions, a background subtraction was necessary in the MS2 channel to a 3-pixel radius, before tracking transcription spots, because of heterogeneous nuclear levels of MCP-GFP.

To measure the Halo::Mam and *E(spl)m*β-MS2/MCP::GFP dynamics in the salivary gland, the intensities of each fluorophore were tracked with the TrackMate plug-in in Fiji. The resulting tracks were imported into MATLAB, where the tracks were sorted according to the time of onset for MS2/MCP detection and the intensities extracted. Pairs of Mam and MS2/MCP foci were then selected on the basis of proximity, with a maximum distance of 1 μm. The tracks were checked for tracking errors and normalized, with 0 and 1 as the lowest and highest intensity values, respectively, because of the large variation in intensity between tracks. The normalized and aligned data were combined to produce average intensities and distances with SEM.

For the measurements of Mam fluctuations in drug treatment experiments, movies were registered in Fiji by using the Mam channel. They were imported into MATLAB to apply Gaussian filtering and bin the signal into a 10-by-10 grid. The regions containing more than 1.5-fold intensity in comparison to the nuclear levels were retained as the ones with persisting intensity over five frames (2.5 min). The maximal levels and standard deviation of each binned region were calculated for each movie, including 15 min for each one.

#### MS2 data processing and parameter calculation

Only MS2/MCP transcription foci tracked for more than five frames were retained, and a maximum gap of two frames was allowed. This produced an initial segregation of active (nuclei ON for at least five frames) and inactive nuclei. Heatmaps were generated for each movie to display only the active nuclei through time. To avoid false negatives arising from nuclei moving out of the field of view, only traces that started after the nuclei had been segmented and finished before their track ends were kept for analysis. Short gaps during ON periods (less than five frames) were interpolated using the mean intensity value of the surrounding frames. The proportion of active nuclei was defined as the percentage of nuclei that come ON per 5-min period in a maximum of 60-min time window per movie. The amplitude was calculated by the alignment of the onset of the ON periods, and values of 0 were assigned when the trace ended to account for the proportion of active nuclei as well as intensities.

For the calculation of active periods, the same procedure was followed, except that the allowable short gaps were extended to fewer than 21 frames (~7 min). Active periods were only calculated when found between two inactive periods. Similarly, inactive periods were quantified when found in between two active ones.

#### MS2 signal calibration

To estimate the average number of mRNA molecules present in MS2 foci from in vivo movies, we first calculated the fluorescence of a single mRNA molecule using a method similar to (*44*, *71*). Briefly, probe sets with different fluorophores for the gene of interest were hybridized to the samples. Then, after applying a threshold to avoid nonspecific particles, the median intensity value of all detected particles was used as a proxy for the intensity of a single particle of mRNA (fig. S2D). ATS total intensities were divided by the intensity obtained for a single particle to determine the number of RNA molecules being transcribed. A *Q*-*Q* plot was then used to relate the intensities of ATSs, in terms of number of molecules from smFISH experiments, to the arbitrary fluorescence intensities of MS2 foci from the same gene (fig. S2E). This yielded a linear relationship with similar parameters for each. The data for the two genes were therefore combined into a single *Q*-*Q* plot, and the parameters obtained from the linear fit were used to provide the conversion factor to determine the number of mRNA molecules transcribed in the MS2 transcription data (fig. S2F).

#### Modeling

Signal deconvolution and kinetic model inference were performed using the BurstDECONV framework (*45*). After calibration, MS2 fluorescence traces for both *E(spl)m7*-MS2 and *E(spl)m*β-MS2 were segmented to isolate only the active periods, thereby excluding prolonged transcriptionally silent periods. This preprocessing step ensured comparability between the two genes, as the inactive periods for *E(spl)m7*-MS2, due to their long durations, were rarely captured within the timescale of our experiments. Each MS2 trace was subsequently deconvolved to infer the timing of individual RNA Pol II initiation events using the parameter settings shown in fig. S7A.

Using the deconvolved initiation events, BurstDECONV computed a survival function describing the distribution of waiting times between consecutive RNA Pol II initiation events. This empirical distribution was subsequently fitted using both biexponential and triexponential models

$$S\left(t\right)=A_{1}e^{\lambda_{1}t}+A_{2}e^{\lambda_{2}t}$$ (1)

$$S\left(t\right)=A_{1}e^{\lambda_{1}t}+A_{2}e^{\lambda_{2}t}+A_{3}e^{\lambda_{3}t}$$ (2)

The minimum number of exponential components required to adequately fit the survival function was selected and interpreted as the number of underlying promoter states.

Model fit quality was evaluated using two complementary approaches. First, confidence intervals for the empirical survival function were calculated using Greenwood’s formula. As an initial validation step, we confirmed whether the best-fit parametric survival curve fell within these confidence bounds. For both *E(spl)m7*-MS2 and *E(spl)m*β-MS2, the two-state model failed this criterion and was therefore excluded from further consideration, while the three-state model satisfied this condition for both genes (fig. S2H). To further assess model accuracy, the Kolmogorov-Smirnov test in combination with the mean squared deviation (used as the objective function) was used as the quantitative metric of goodness of fit. Our analysis supports a nonsequential three-state model (Fig. 2J) but does not exclude a sequential alternative, where the promoter transitions obligatorily through the transient OFF1 state to move between the ON and long OFF2 states. However, this sequential arrangement implies a strong dependence between the two inactive states, a relationship for which a plausible mechanistic basis is lacking.

The multiexponential distribution fits were used to infer kinetic parameters for Markovian models of transcriptional bursting under steady-state conditions. The resulting kinetic estimates are provided in fig. S2J, alongside the corresponding objective function values and Kolmogorov-Smirnov test statistics. We note that *k*_ini_, the parameter showing the greatest difference between *E(spl)m7* and *E(spl)m*β, was also the most reliably inferred in method validation using synthetic datasets (*45*).

Preliminary estimates of the decay constants and population proportions (the λ and A parameters in Eqs. 1 and 2) were obtained using a computationally efficient and numerically stable algorithm on the basis of solving a linear system derived from cumulative integrals (https://github.com/juangburgos/FitSumExponentials/tree/main). These estimates were subsequently used to initialize the linear regression procedure in BurstDECONV, substantially accelerating the fitting process.

As reliable extraction of kinetic parameters required that the system had reached a steady-state regime, we computed the average interval between successive Pol II initiation events (⟨τ⟩) within a narrow, temporal window. This average interval has been shown to be proportional to the reciprocal of the product of pON (the likelihood of the promoter being in Prom ON state) and *k*_ini_, the initiation rate during the active state (*72*, *73*). Hence, stability in ⟨τ⟩ across different windows served as an indicator that the underlying kinetic parameters remained unchanged over time. A window width of four frames (corresponding to 80 s) was chosen, which provided enough initiation events to yield a robust estimate of ⟨τ⟩. To assess the precision of these estimates, we applied a bootstrap resampling approach to derive 95% confidence intervals for both genes under investigation (fig. S7B).

#### Statistical analysis

Two-tailed *t* test was applied in cases with *n* > 30 for both conditions tested. Otherwise, normality was checked via a Shapiro-Wilk test. When one or more of the samples were not normal, a Wilcoxon rank sum test was applied. When more than three groups were compared, an analysis of variance (ANOVA) test was carried out. In all cases, significance was presented as *P < 0.05, **P < 0.01, ***P < 0.001, and ****P < 0.0001, and *P* values are provided in figure legends.
