# Supplementary material for: Direct observation of Notch signaling–induced transcription hubs mediating gene-expression responses
Source: Sci Adv. 2026 Mar 6;12(10):eaea5664. doi: 10.1126/sciadv.aea5664 (PMC12965292; doi:10.1126/sciadv.aea5664)
Supplement: Supplementary file 1 — Figs. S1 to S7 Tables S1 to S3 Legends for movies S1 to S6 [file sciadv.aea5664_sm.pdf]

Science Advances
AAAS

Supplementary Materials for

Direct observation of Notch signaling–induced transcription hubs mediating gene-expression responses

Carmen Santa-Cruz Mateos *et al.*

Corresponding author: Sarah Bray, sjb32@cam.ac.uk

Sci. Adv. **12**, eaea5664 (2026)
DOI: 10.1126/sciadv.aea5664

The PDF file includes:

Figs. S1 to S7
Tables S1 to S3
Legends for movies S1 to S6

Other Supplementary Material for this manuscript includes the following:

Movies S1 to S6

Figure S1

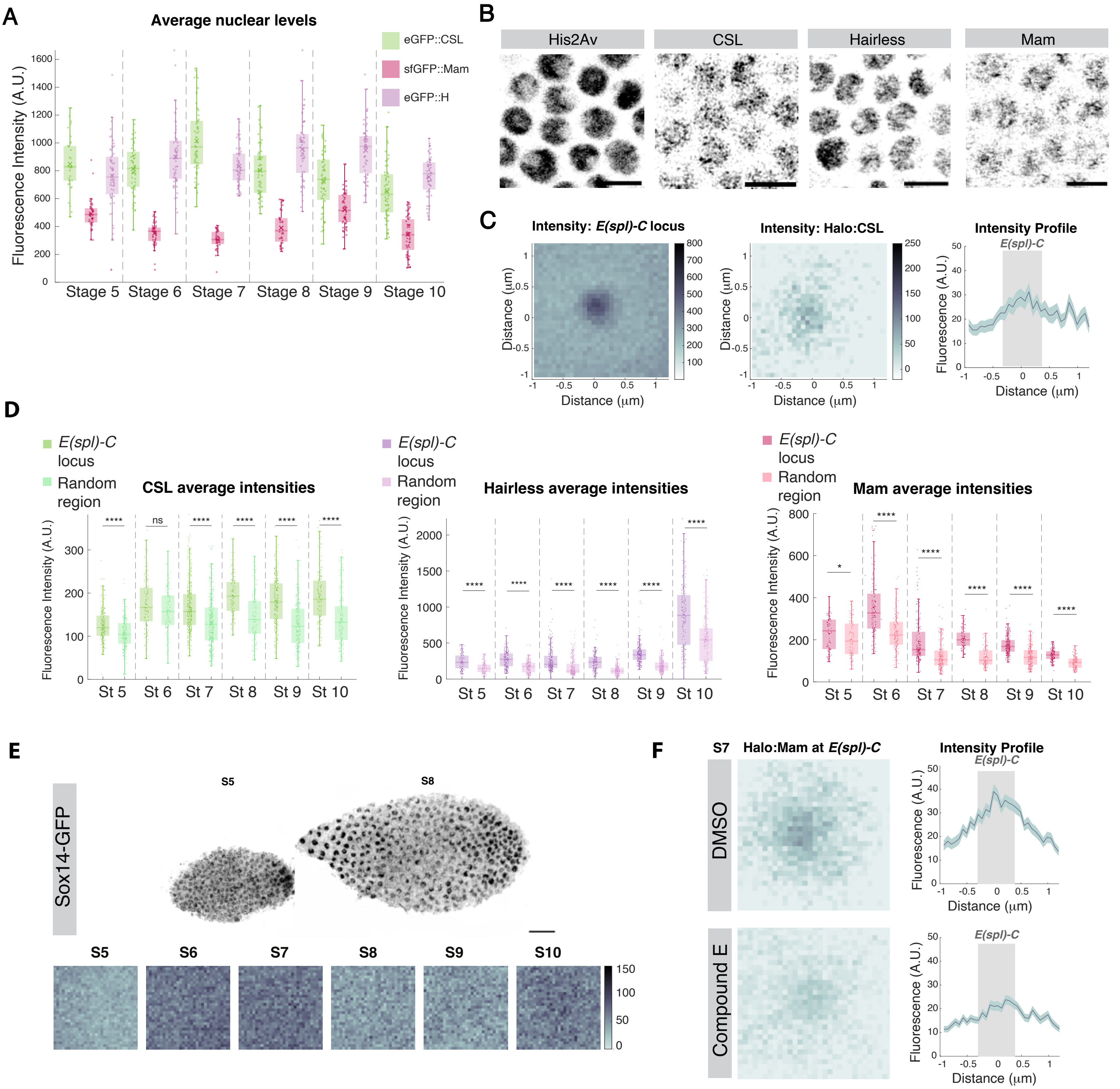

Figure S1.Nuclear levels remain constant during the period of Mam enrichment at E(spl)-C locus which requires Notch activity.

(A) Nuclear levels of eGFP:CSL, eGFP:H and sfGFP:Mam remain the same throughout stages 5-9 development. N= 36, 61, 61, 61, 61, 61 (eGFP:CSL), 61, 59, 61, 61, 61, 61 (eGFP:H) and 46, 53, 37, 37, 57 and 57 (sfGFP:Mam). **(B)** Live Images from different samples illustrating heterogeneity of CSL, Hairless and Mam, as opposed to Histones homogeneous pattern in nuclei. Scale bar represents 5 µm. **(C)** Example, using CSL::Halo data and image from Figure 1E S6, illustrating pipeline to quantify enrichment at *E(spl)-C* locus. Images are centered with respect to the tagged *E(spl)-C* locus (left) and the intensity values obtained from the aligned images of the fluorescent transcription factor are averaged and normalized to create an average intensity pixel map (right, image as in Figure 1E). **(D)** Enrichment levels of CSL, Hairless and Mam at *E(spl)-C* compared to a random region. **(E)** Expression of Sox14-GFP in stage 5 and 8 (upper panel). Scale bar = 20µm. Average intensity of Sox14-GFP at *E(spl)-C* during stages 5 to 10 shows no enrichment. n = 70, 123, 122, 78, 72 and 95, respectively. **(F)** Effects of Notch inhibition on Mam enrichments. Average pixel plots and intensity profiles as in A with average Mam intensities at *E(spl)-C* in control (DMSO, upper panels) and γ-secretase (Compound E) treated egg chambers (lower panels). Grey shading indicates *E(spl)-C* locus. n= 198 (DMSO), 194 (Compound E).

Figure S2

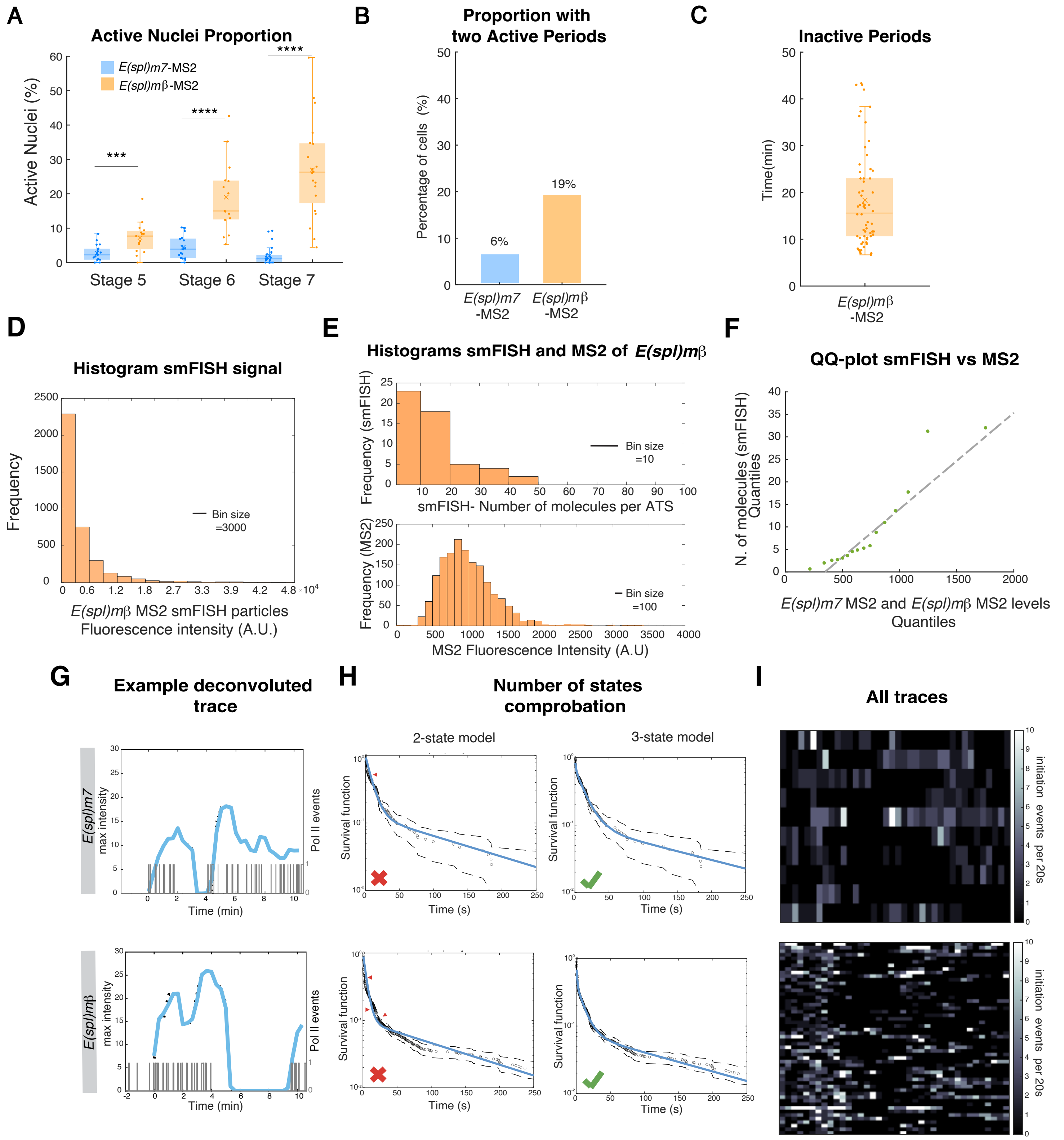

J

	k1+	k1-	k2+	k2-	kini	p1	p2	p3	Obj	K-S test p-value
E(spl)m7	6.56e-3	2.94e-2	1.77e-1	4.10e-1	**3.04e-1**	5.75e-1	2.97e-1	1.28e-1	5.00e-3	9.99e-1
E(spl)mβ	7.02e-3	5.11e-2	1.40e-1	3.26e-1	**6.52e-1**	6.86e-1	2.20e-1	9.42e-2	1.06e-2	9.99e-1

Figure S2. Calibration of MS2/MCP intensities and results from modelling promoter states.

(A) Proportions of nuclei actively transcribing *E(spl)m7*-MS2 (blue) and *E(spl)mβ*-MS2 (orange) at the stages indicated using smFISH. **(B)** Example of calibration process. Frequency histogram of particle intensities from an smFISH image labelled with *E(spl)mβ*-670.**(C)** Histograms of ATS calibrated signals from *E(spl)mβ*-670 smFISH images (X-axis is number of molecules, upper graph) and intensities of E(spl)mβ-MS2 transcription foci (lower graph). **(D)** Percentage of active nuclei showing two or more ON periods in their traces of MS2 activity. **(E)**Time between two Active periods from *E(spl)mβ*-MS2 (orange) traces containing >1 active period. **(F)** Combined Q-Q plot of number of molecules in ATS from smFISH

images with respect to MS2 intensities from a representative movie. **(G)** Examples of MS2 transcription profiles (black dashed lines) with results from BurstDECONV analysis; inferred profile (blue) and inferred PolII initiation events (black lines). **(H)** Graphs testing fit to 2-state or 3-state promoter models. Red arrowheads point to parametric survival curve not fitting in the confidence intervals. **(I)** Heat map of PolII initiation events assigned to transcription tracks from *E(spl)m7*-MS2 and *E(spl)m*β-MS2.**(J)** Table showing the resultant values from the modelling. The biggest differences found between *E(spl)m7*-MS2 and *E(spl)m*β-MS2 is kini.

Figure S3

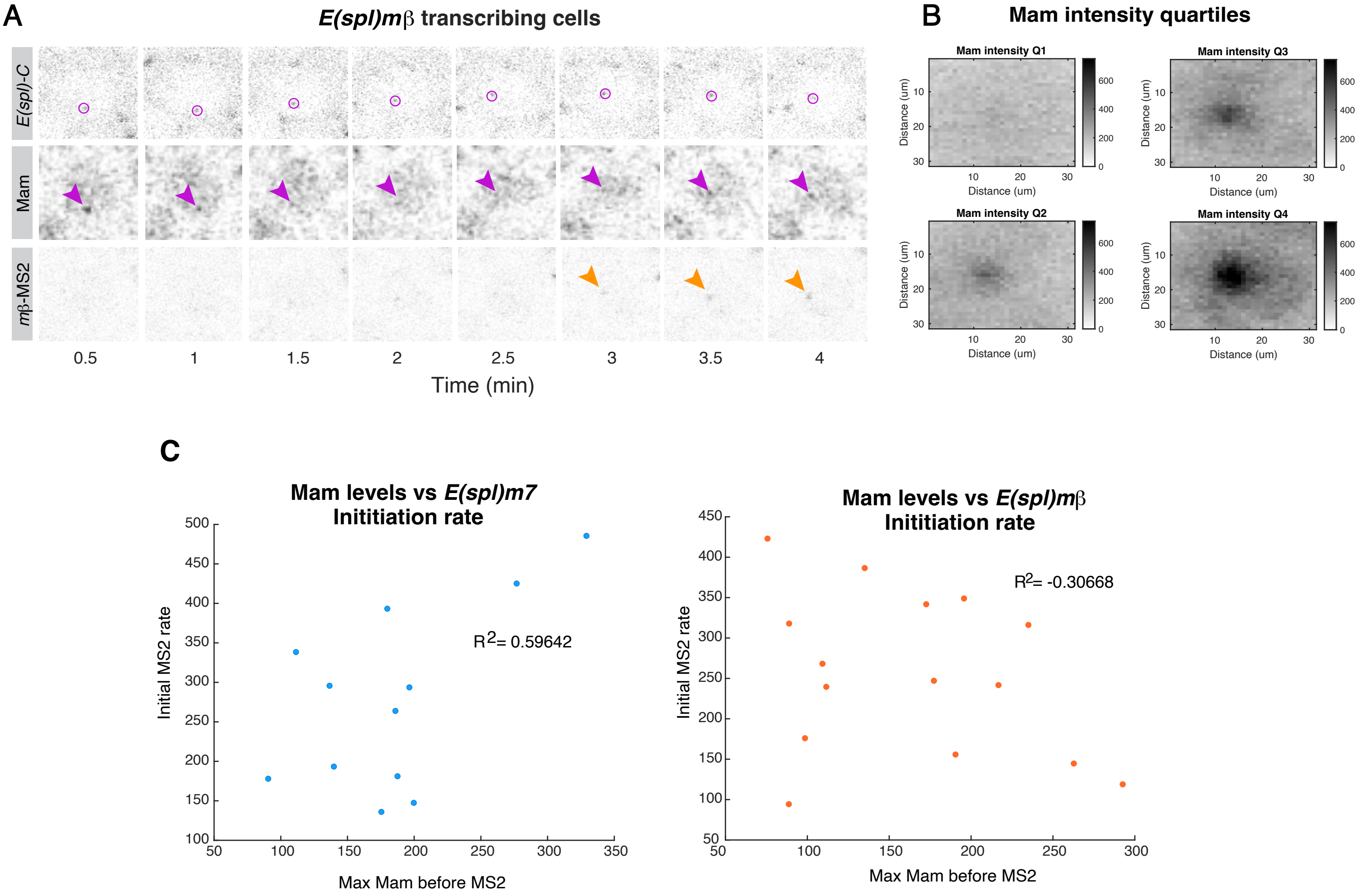

Figure. S3. Variations in Mam enrichment levels.

(A) Confocal images from *in vivo* movie (Supplementary movie S4), tracking Mam-Halo and *E(spl)m*β-MS2 in relation to *E(spl)-C* locus (magenta circle). Mam enrichment (arrowhead) precedes *E(spl)m*β-MS2 transcription (orange arrowhead). Scale bar represents 2 μm. **(B)** Average pixel intensities of Mam enrichment at *E(spl)-C* in St 6 nuclei from Figure 1D,3B have been partitioned into quartiles which reveals variations in the levels of enrichment (n= 141). **(C)** Correlation between maximal Mam intensity values and PolII loading rate, inferred from the initial slope of *E(spl)m7*-MS2 transcription profile (R2 = 0.596) and *E(spl)m*β-MS2 (R2=-0.306).

Figure S4

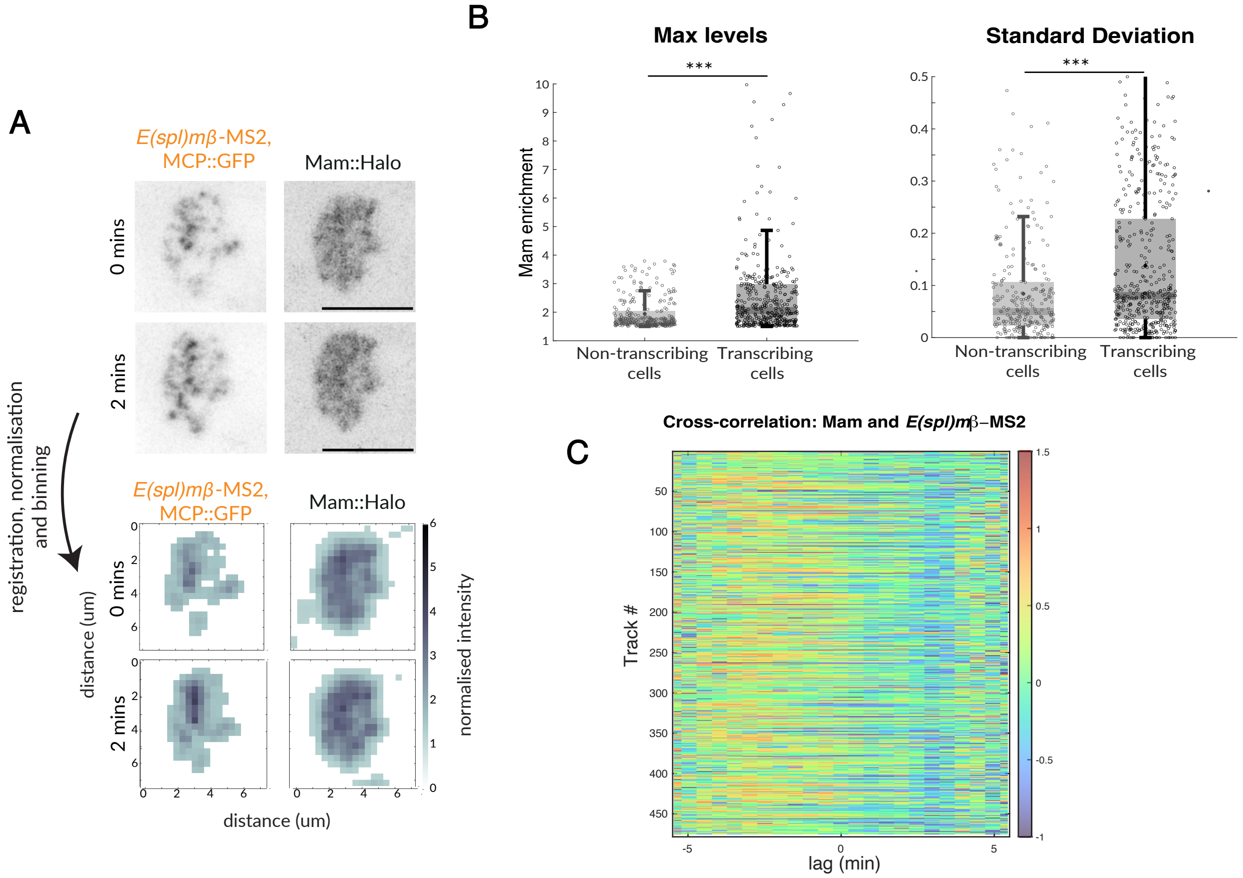

Figure S4: Analysis pipeline for Mam enrichment and transcription profiles in salivary gland nuclei.

(A) Cartoon illustrating analysis pipeline to measure intensities of *E(spl)m*β-MS2 foci and condensed Mam hubs in salivary gland nuclei. Aligned Images were averaged and intensities plotted respect to time. For further quantifications, pixel intensities were binned using a 10 x 10 grid. Scale bars represent 5 μm. **(B)** Boxplots with maximum levels and standard deviations of binned Mam intensities within *E(spl)-C* in non-transcribing and transcribing nuclei. **(C)** Heatmap of cross-correlation between paired *E(spl)m*β–MS2 and Mam tracks. Transcription onset is centered (0) and time-lags were applied to Mam tracks as indicated and the correlation calculated, turbo shading indicates strength of correlation.

Figure S5

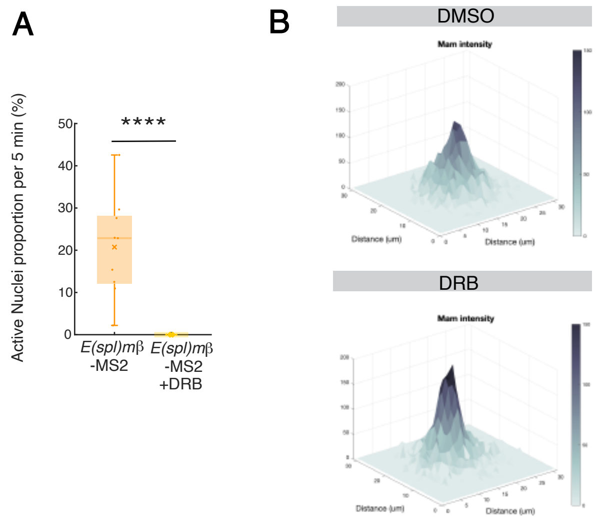

Figure S5: Transcription inhibition with DRB stabilizes Mam hub.

(A-B) Effect of DRB treatment on transcription and Mam enrichment in follicle cells. **(A)** Average proportions of transcribing nuclei using *E(spl)m*β-MS2 from control (DMSO, n=10) and DRB treated (DRB, n=10) stage 6 egg chambers. **(B)** 3D plot (left panels) of Mam mean intensity at *E(spl)-C* in control (DMSO, n=363) and DRB treated (DRB, n=388) tissues as in A, with intensities partitioned into quartiles (right panels).

Figure S6

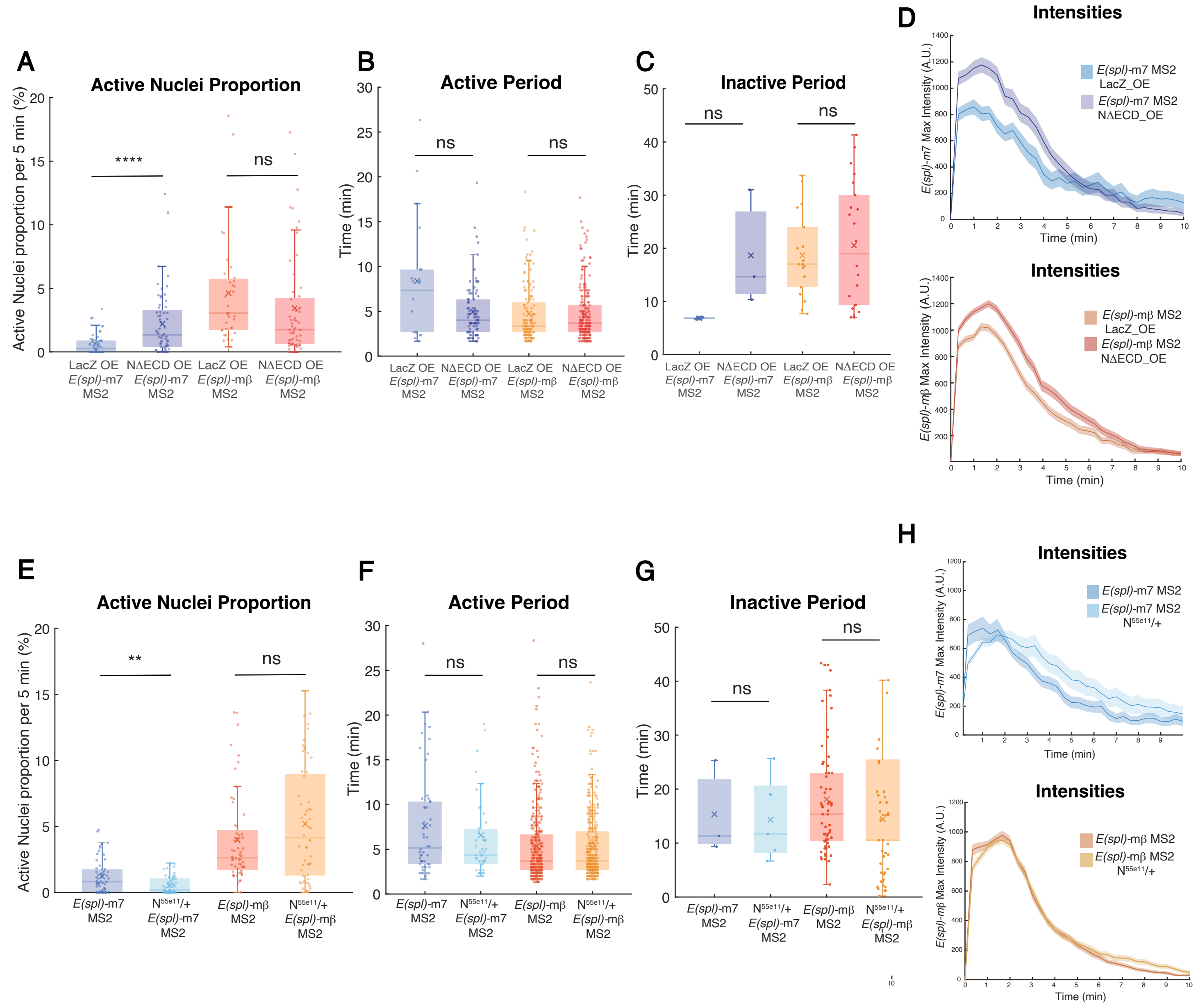

Fig. S6. smFISH experiments confirm changes of transcription output when Notch dose is altered.

(A) Boxplots of active nuclei proportions from live imaging of *E(spl)m7*-MS2 and *E(spl)m*β-MS2 in control and mild *NΔECD* overexpression. Duration of active periods are similar for all conditions as indicated. **(B)** Duration of active periods for all conditions.**(C)**Duration of inactive periods for all conditions as indicated. **(D)** Transcription amplitude profiles; mean track intensities from *E(spl)m7*-MS2 (blue, left) and *E(spl)m*β -MS2 (orange, right) in control (mid-blue/orange) and Notch overexpression (dark blue/orange) conditions. **(E)** Boxplots of active nuclei proportions from live imaging of *E(spl)m7*-MS2 and *E(spl)m*β-MS2 in control and *N55e11/+*. **(F)** Duration of active periods for all conditions. **(G)** Duration of inactive periods for all conditions as indicated. **(H)** Transcription amplitude profiles; mean track intensities from *E(spl)m7*-MS2 (blue, left) and *E(spl)m*β-MS2 (orange, right) in control (mid-blue/orange) and Notch heterozygotes (light blue/orange) conditions. Boxplots indicate median, with 25–75 quartiles; error bars are SD. In D,I graphs, SEM is represented by shading.

Figure S7

A

	E(spl)m7	*E(spl)mβ*
**RNA Pol II speed (bp/s)**	45	45
**Time Resolution (s)**	20	20
**Min Pol II distance (bp)**	30	30
**mRNA length pre MS2 (bp)**	679	0
**MS2 length (bp)**	1330	1330
**mRNA length post MS2 (bp)**	3128	4106

B

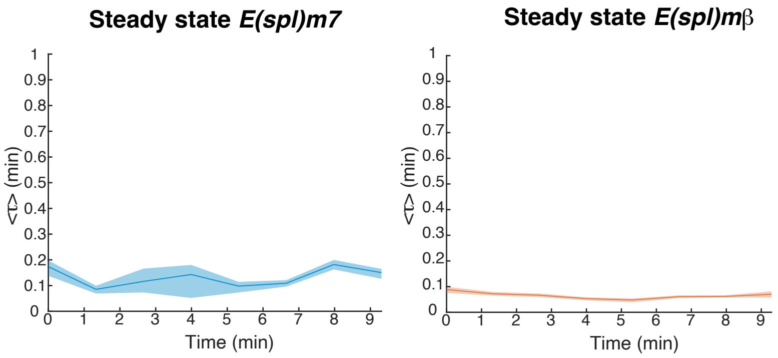

Figure S7: Parameters and Steady state confirmation for modelling.

(A) Parameters feeded to the BURSTDECONV model for *E(spl)m7*-MS2 and*E(spl)m*β-MS2. **(B)** Average interval between successive Pol II initiation events (<τ>) through a 4 frames time window for *E(spl)m7*-MS2 and*E(spl)m*β-MS2 confirmed that steady state has been reached stage 6.

Table S1: Details of Drosophila lines used.

Line / Element	Genotype (FlyBase Nomenclature)	Source
Delta::mScarlet-I	*TI{TI}Delta[mScarlet-I]*	[71]
Locus tag *(E(spl)-C locus + ParB1)*	*TI{P{w[+]=intA}3xP3-RFP.attP}E(spl)mδ-HLH, P{w[+]=UAS-ParA::mCherry}attP86Fb / TM6B*	[16]
GFP::CSL	*M{ w[+]=Su(H)::EGFP}attP86Fb*	[15]
Halo::CSL	*M{ w[+]=Su(H)::Halo}attP86F*	[15]
GFP::Hairless	*M{ w[+]=H.WT.EGFP}51D*	[16]
Halo::Mam	*TI{TI}mamRA[Halo]*	[15]
GFP::Mam	*TI{TI}mamRA[sfGFP]*	[15]
Sox14::GFP	*w[1118]; PBac{y[+mDint2] w[+mC]=Sox14-GFP.FPTB}*	BDSC #55842
E(spl)m7-MS2	*TI{24xMS2-lacZ-SV40}E(spl)m7-HLH*	This paper
E(spl)mβ-MS2	*TI{24xMS2-lacZ-SV40}E(spl)mβ-HLH*	[71]
hsp83-MCP::GFP	*P{w[+mC]=Hsp83-MCP-GFP}3*	BDSC #7280
His2Av::RFP	*P{His2Av-mRFP}*	BDSC #23650
UAS-NΔECD	*P{w[+]=UAS-NotchΔECD}*	[16]
UAS-NΔECD::mCherry	*P{w[+]=UAS-NotchΔECD:*mCherry *}*	This paper
tj-GAL4	*w[*], P{w[+]=GawB}Tj*	St Johnston lab
tj-GAL4::tub-$Gal80^{ts}$	*w[*], P{w[+]=GawB}Tj , P{w[+]=tubP-GAL80[ts]}*	St Johnston lab
UAS-LacZ	*P{w[+]=UAS-nls-LacZ}*	Brand lab
hs-Flp	*P{ry[+t7.2]=hsFLP}1, y[1] w[1118]; Dr[1]/TM3, Sb[1]*	BDSC #26902
Tub>FRT.STOP.FRT>GAL4::UAS-mTandemTomato	*w[1118]* ; P{*tubP[FRT.CD2-Stop] GawB}*, 10x*UAS-IVS-myr::tdTom in attP40* / CyO,P{w[+mC]=Dfd-GMRYFP}2	Van den Ameele lab
N^{55e11} *FRT19A*	*w[1118] N[55e11] P{ry[+t7.2]=neoFRT}19A*	BDSC #28813
yw	*Y[1] w[1118]*	BDSC #6598

Table S2: Detailed genotypes for each Figure.

Figure	X chromosome	II chromosome	III chromosome
Figure 1B	*w*	*Sco/CyO*	*Delta-$^{mScarlet-I}$*
Figure 1D	*w*	*tj-Gal4*	*Halo::CSL, E(spl)-C[m∂ intA}, UAS-ParA::mCherry*
Figure 1D	*w*	*tj-Gal4/GFP::Hairless*	*E(spl)-C[m∂ intA}, UAS-ParA::mCherry*
Figure 1D	*w*	*tj-Gal4/Halo::Mam*	*E(spl)-C[m∂ intA}, UAS-ParA::mCherry*
Figure 1E	As figure 1D		
Figure 2A,B	*yw*		
Figure 2D	*w*	*hsp83-MCP::GFP*	*E(spl)mβ-MS2/ His2Av-RFP*
Figure 2E,F,G,H	*w*	*hsp83-MCP::GFP*	*E(spl)mβ-MS2/ His2Av-RFP*
Figure 2E,F,G,H	*w*	*hsp83-MCP::GFP*	*E(spl)m7-MS2/ His2Av-RFP*
Figure 3A	*w*	*tj-Gal4/Halo::Mam*	*E(spl)-C[m∂ intA}, UAS-ParA::mCherry*
Figure 3B,C,F	*w*	*hsp83-MCP::GFP, tj-Gal4/Halo::Mam*	*E(spl)m7-MS2/ E(spl)-C[m∂ intA}, UAS-ParA::mCherry*
Figure 3D,E,G	*w*	*hsp83-MCP::GFP, tj-Gal4/Halo::Mam*	*E(spl)mβ -MS2/ E(spl)-C[m∂ intA}, UAS-ParA::mCherry*
Figure 4	*1151-Gal4*	*hsp83-MCP::GFP /Halo::Mam*	*E(spl)mβ -MS2/ UAS-NΔECD*
Figure 5A	*w*	*tj-Gal4/Halo::Mam*	*E(spl)-C[m∂ intA}, UAS-ParA::mCherry*
Figure 5B,C,E	*1151-Gal4*	*hsp83-MCP::GFP /Halo::Mam*	*E(spl)mβ -MS2/ UAS-NΔECD*
Figure 6A,B	*w*	*tj-GAL4::tub-Gal80^{ts}/Halo::Mam*	*E(spl)-C[m∂ intA}, UAS-ParB::GFP/ UAS-NΔECD::mCherry*
Figure 6A,B	*w*	*tj-GAL4::tub-Gal80^{ts}/Halo::Mam*	*E(spl)-C[m∂ intA}, UAS-ParB::GFP/ UAS-LacZ*
Figure 6C,D	*w*	*tj-GAL4::tub-Gal80^{ts}*	*UAS-NΔECD::mCherry*
Figure 6C,D	*w*	*tj-GAL4::tub-Gal80^{ts}*	*UAS-LacZ*
Figure 6F,G	*N^{55e11} FRT19A*	*tj-Gal4/Halo::Mam*	*E(spl)-C[m∂ intA}, UAS-ParA::mCherry*

Figure	X chromosome	II chromosome	III chromosome
Figure 6F,G	*w*	tj-Gal4/*Halo::Mam*	*E(spl)-C[m∂ intA}, UAS-ParA::mCherry*
Figure 6H,I	*N^{55e11} FRT19A*		
Figure 6H,I	yw		
Figure S1A	*w*		*GFP::CSL*
Figure S1A		*GFP::Hairless*	
Figure S1A		*GFP::Mam*	
Figure S1B	*w*		*His2Av-RFP*
Figure S1B	*w*	tj-Gal4	*Halo::CSL, E(spl)-C[m∂ intA}, UAS-ParA::mCherry*
Figure S1B	*w*	tj-Gal4/*GFP::Hairless*	*E(spl)-C[m∂ intA}, UAS-ParA::mCherry*
Figure S1B	*w*	tj-Gal4/*Halo::Mam*	*E(spl)-C[m∂ intA}, UAS-ParA::mCherry*
Figure S1C		tj-Gal4	*Halo::CSL, E(spl)-C[m∂ intA}, UAS-ParA::mCherry*
Figure S1E	*w*	tj-Gal4/*Sox14::GFP*	*E(spl)-C[m∂ intA}, UAS-ParA::mCherry*
Figure S1F	*w*	tj-Gal4/*Halo::Mam*	*E(spl)-C[m∂ intA}, UAS-ParA::mCherry*
Figure S3A	*w*	*hsp83-MCP::GFP,* tj-Gal4/*Halo::Mam*	*E(spl)mβ -MS2/ E(spl)-C[m∂ intA}, UAS-ParA::mCherry*
Figure S3B	*w*	tj-Gal4/*Halo::Mam*	*E(spl)-C[m∂ intA}, UAS-ParA::mCherry*
Figure S4	*1151-Gal4*	*hsp83-MCP::GFP /Halo::Mam*	*E(spl)mβ -MS2/ UAS-NΔECD*
Figure S5A, B,C,D	*w*	tj-Gal4/*Halo::Mam*	*E(spl)-C[m∂ intA}, UAS-ParA::mCherry*
Figure S5E,F	*1151-Gal4*	*hsp83-MCP::GFP /Halo::Mam*	*E(spl)mβ -MS2/ UAS-NΔECD*
Figure S6A,B,C,D	*hs-Flp*	*Tub>FRT.STOP.FRT> GAL4::UAS-mTandemTomato/ hsp83-MCP::GFP*	*E(spl)m7 -MS2/ UAS-NΔECD::mCherry*

**Figure**	**X chromosome**	**II chromosome**	**III chromosome**
Figure S6A,B,C,D	*hs-Flp*	*Tub>FRT.STOP.FRT> GAL4::UAS-mTandemTomato/ hsp83-MCP::GFP*	*E(spl)m7 -MS2/ UAS-LacZ*
Figure S6A,B,C,D	*hs-Flp*	*Tub>FRT.STOP.FRT> GAL4::UAS-mTandemTomato/ hsp83-MCP::GFP*	*E(spl)mβ -MS2/ UAS-NΔECD::mCherry*
Figure S6A,B,C,D	*hs-Flp*	*Tub>FRT.STOP.FRT> GAL4::UAS-mTandemTomato/ hsp83-MCP::GFP*	*E(spl)mβ -MS2/ UAS-LacZ*
Figure S6E,F,G,H	N^{55e11} *FRT19A*	*hsp83-MCP::GFP*	*E(spl)m7-MS2/ His2Av-RFP*
Figure S6E,F,G,H	w	*hsp83-MCP::GFP*	*E(spl)m7-MS2/ His2Av-RFP*
Figure S6E,F,G,H	N^{55e11} *FRT19A*	*hsp83-MCP::GFP*	*E(spl)mβ-MS2/ His2Av-RFP*
Figure S6E,F,G,H	w	*hsp83-MCP::GFP*	*E(spl)mβ-MS2/ His2Av-RFP*

Table S3: results of statistical analysis of data presented in Figures

Figure	Experiment	n	p Value
Figure 1D	CSL. St 5, 6, 7, 8, 9, 10	n=221, 137 377,137,259, 158	
Figure 1D	Mam. St 5, 6, 7, 8, 9, 10	n=92, 141, 173, 171, 287, 219	
Figure 1D	Hairless. St 5, 6, 7, 8, 9, 10	n= 94, 186, 241, 158, 248, 207	
Figure S1A	CSL. St 5, 6, 7, 8, 9, 10	n= 36, 61, 61, 61, 61, 61	P=0
Figure S1A	Hairless. St 5, 6, 7, 8, 9, 10	n=61, 59, 61, 61, 61, 61	P= 4.664e-11
Figure S1A	Mam. St 5, 6, 7, 8, 9, 10	n=46, 53, 37, 37, 57,57	P=0
Figure S1D	CSL. St 5, 6, 7, 8, 9, 10	n=221, 137 377,137,259, 158	P= 7.51E-08, 0.06, 2.05E-11, 1.84E-14, 4.85E-26, 2.30E-13
Figure S1D	Mam. St 5, 6, 7, 8, 9, 10	n=92, 141, 173, 171, 287, 219	P= 0.03, 1.82E-15, 2.26E-12, 1.23E-26, 1.11E-27, 1.11E-19
Figure S1D	Hairless. St 5, 6, 7, 8, 9, 10	n= 94, 186, 241, 158, 248, 207	P= 2.321E-10, 1.47E-21, 2.58E-12, 1.86E-22, 4.34E-49, 1.78E-17
Figure 2B	Active nuclei *E(spl)m7 vs E(spl)mβ* stage 6	n=20, 18	p=4,5e-10
Figure 2G	Active nuclei *E(spl)m7-MS2 vs E(spl)mβ–MS2*	n=57, 70	p=3.16e-9
Figure 2H	Active periods *E(spl)m7-MS2 vs E(spl)mβ–MS2*	n=54,342	p = 0.00114
Figure 2I	Inactive periods *E(spl)m7-MS2 vs E(spl)mβ–MS2*	n=3,63	P=0.7464
Figure S2A	Active nuclei *E(spl)m7 vs E(spl)mβ* stages 5,6 & 7	n=20, 16, 20,18,24,21	p=9.82E-4, 6.89E-7, 2.4E-10
Figure S2D	Inactive periods *E(spl)m7-MS2 vs E(spl)mβ–MS2*	n=3,63	P=0.7464
Figure 3A	3D Mam stage 6	n=141. 3 e.c.	
Figure 3C	Mam with *E(spl)m7-MS2* in transcribing cells	n=12. 7 e.c.	

Figure	Experiment	n	p Value
Figure 3D	Mam with *E(spl)mβ*–MS2 in transcribing cells	n=15. 5 e.c.	
Figure 3E	Mam with *E(spl)mβ*–MS2 in no transcribing cells	n=7. 4 e.c.	
Figure 3F	Cross-correlation Mam with *E(spl)m7-MS2*	n=12	On figure.
Figure 3G	Cross-correlation Mam with *E(spl)mβ*–MS2	n=15	On figure.
Figure S3B	Quartiles Mam stage 6	n=141. 3 e.c.	
Figure S3C	Correlation Mam with *E(spl)m7-MS2* slope	n=12	On figure.
Figure S3C	Correlation Mam with *E(spl)mβ–MS2* slope	n=15	On figure.
Figure 4C	Mam with *E(spl)mβ*–MS2 in transcribing cells. SGs	n=20	
Figure 4D	Cross-correlation Average Mam with *E(spl)mβ*–MS2	n=20, (435 regions)	On figure.
Figure 4E	3D plot transcribing and no transcribing cells. SGs.	n=7, 10	
Figure 4F	Mam autocorrelation in transcribing and no transcribing cells. SGs.	n=7,10	On figure.
Figure S4B	Levels of Mam and SD in no transcribing and transcribing cells. SGs.	n=10 (276 regions), 7 (435 regions)	
Figure S4C	Cross-correlation Average Mam with *E(spl)mβ*–MS2.	n=20, (435 regions)	On figure.
Figure 5A	3D Mam in DMSO vs.Triptolide	n= 531, 540. 7, 6 e.c.	
Figure 5B	Active nuclei proportion of *E(spl)m7-MS2* before vs after triptolide	n=3, 3	p= 4.22e-5
Figure 5C	3D Mam and *E(spl)mβ*–MS2 transcription in DMSO vs.Triptolide	n=9 (327 regions), 6 (345 regions)	

Figure	Experiment	n	p Value
Figure 5D	3D Mam and *E(spl)mβ*–MS2 in DMSO vs. A485	n=9 (327 regions), 6 (191 regions)	
Figure S5A	Active nuclei proportion of *E(spl)mβ–MS2* in control vs DRB.	n=10, 10	p= 8.86e-5
Figure S5B	3D Mam in DMSO vs.DRB	n=363, 388	
Figure 6B	3D Mam in Control and *NΔECD*	n=240, 279 from 5, 6 e.c.	
Figure 6D	Active Nuclei proportion *E(spl)m7* control *vs NΔECD*	n= 4, 5	p= 0.039
Figure 6D	Active Nuclei proportion *E(spl)mβ* control *vs NΔECD*	n= 4, 5	p=0.44
Figure 6F	3D Mam in Control and N^{55e11}/+	n=137, 76	
Figure 6H	Active Nuclei proportion *E(spl)m7* control *vs* N^{55e11}/+	n=15, 9	p=0.031
Figure 6H	Active Nuclei proportion *E(spl)mβ* control *vs* N^{55e11}/+	n=11,9	p=0.062
Figure S6A	Active Nuclei proportion *E(spl)m7-MS2* control *vs NΔECD*	n= 5 , 5 e.c.	p=7.88e-6
Figure S6A	Active Nuclei proportion *E(spl)mβ–MS2* control *vs NΔECD*	n=3, 5 e.c.	p=0.18
Figure S6B	Active periods *E(spl)m7-MS2* control *vs NΔECD*	n= 18, 84	p= 0.1185
Figure S6B	Active periods *E(spl)mβ–MS2* control *vs NΔECD*	n= 134, 211	p = 0.802
Figure S6C	Inactive periods *E(spl)m7-MS2* control *vs NΔECD*	n=2, 3	p = 0.2
Figure S6C	Inactive periods *E(spl)mβ–MS2* control *vs NΔECD*	n=18, 22	p = 0.92

Figure	Experiment	n	p Value
Figure S6D	Intensities *E(spl)m7-MS2* control *vs NΔECD*	n=64 tracks from 5 e.c., 85 tracks from 5 e.c.	
Figure S6D	Intensities *E(spl)mβ–MS2* control *vs NΔECD*	n=155 tracks from 3 e.c., 231 tracks from 5 e.c.	
Figure S6E	Active Nuclei proportion *E(spl)m7-*MS2 control *vs* N^{55e11}/+	n=5, 6 e.c.	p = 7.88e-6
Figure S6E	Active Nuclei proportion *E(spl)mβ–MS2* control *vs* N^{55e11}/+	n= 6, 5 e.c	p = 0.184
Figure S6F	Active periods *E(spl)m7-MS2* control *vs* N^{55e11}/+	n=52, 47	p = 0.381
Figure S6F	Active periods *E(spl)mβ–MS2* control *vs* N^{55e11}/+	n=340, 326	p = 0.649
Figure S6G	Inactive periods *E(spl)m7-MS2* control *vs* N^{55e11}/+	n=3, 5	p = 4.215e-8
Figure S6G	Inactive periods *E(spl)mβ–MS2* control *vs* N^{55e11}/+	n=63, 45	p = 0.227
Figure S6H	Intensities *E(spl)m7-MS2* control *vs* N^{55e11}/+	n= 66 tracks from 5 e.c., 35 tracks from 6e.c.	
Figure S6H	Intensities *E(spl)mβ–MS2* control *vs* N^{55e11}/+	n=443 from 6 e.c., 318 from 5 e.c.	

Other Supplementary Materials for this manuscript include the following:

Movie S1: Live imaging of E(spl)m7-MS2/MCP::GFP transcription, stage 6.
Movie S2: Live imaging of E(spl)mβ-MS2/MCP::GFP transcription, stage 6.
Movie S3: 3 colour live imaging of E(spl)-locus (yellow), Mam::Halo (magenta), E(spl)m7-MS2/MCP::GFP transcription (blue), stage 6.
Movie S4: 3 colour live imaging of E(spl)-locus (yellow), Mam::Halo (magenta), E(spl)mβ-MS2/MCP::GFP transcription (orange), stage 6.
Movie S5: Live imaging of E(spl)mβ-MS2/MCP::GFP transcription (green in overlay) and Mam::Halo (magenta in overlay), salivary gland nucleus.
Movie S6: 3D plots from live imaging of Mam::Halo and E(spl)mβ-MS2/MCP::GFP transcription in transcribing and non-transcribing salivary gland nuclei.
